# Coordinated TNF–NF‐κB and PI3K–Akt Modulation by Yueju Pill in Hashimoto's Thyroiditis: A Network Pharmacology and Molecular Docking Study

**DOI:** 10.1002/cbdv.71790

**Published:** 2026-09-30

**Authors:** Hao Zhang, Kuojun Ren, Shengying Wang

**Affiliations:** ^1^ The First Clinical Medical College Anhui University of Chinese Medicine Hefei Anhui China; ^2^ Department of Thyroid and Breast Surgery The First Affiliated Hospital of Anhui University of Chinese Medicine Hefei Anhui China; ^3^ Breast Cancer Center The First Affiliated Hospital of University of Science and Technology of China Division of Life Sciences and Medicine University of Science and Technology of China Hefei Anhui China; ^4^ Breast Cancer Center Anhui Provincial Cancer Hospital Hefei Anhui China

**Keywords:** dual‐axis core model, Hashimoto's thyroiditis, molecular docking, network pharmacology, Yueju pill

## Abstract

To predict the polypharmacological mechanism of Yueju pill (YJP) against Hashimoto's thyroiditis (HT), this study integrated network pharmacology and molecular docking to develop a computationally derived “dual‐axis core” hypothesis involving the TNF–NF‐κB inflammatory axis and the PI3K–Akt survival/apoptosis axis. Candidate active compounds and putative targets were identified, followed by PPI network construction and GO and KEGG enrichment analyses. Representative compounds were further evaluated by molecular docking, with redocking and reference–ligand comparisons used to assess docking reliability. A total of 167 common drug–disease targets were identified, from which 13 hub targets were screened. Enrichment analysis highlighted the TNF, PI3K–Akt, IL‐17, Th17 cell differentiation, and apoptosis‐related pathways. Quercetin, luteolin, kaempferol, and wogonin showed predicted interactions with multiple inflammation‐ and apoptosis‐related targets. Redocking reproduced crystallographic binding modes for CASP3, IKBKB, MAPK1, and AKT1 with RMSD values below 2.0 Å. Overall, the integrated computational analyses support a testable “dual‐axis core” hypothesis in which YJP may coordinately modulate inflammatory and survival/apoptosis‐related signaling in HT, providing prioritized targets and a mechanistic framework for future experimental validation.

## Graphical abstract

Network pharmacology and molecular docking identify a coordinated dual‐axis regulatory framework for Yueju pill in Hashimoto's thyroiditis. Candidate compounds and prioritized targets converge on TNF–NF‐κB inflammatory and PI3K–Akt survival/apoptosis signaling, providing computational support for a testable multi‐target hypothesis requiring experimental validation.

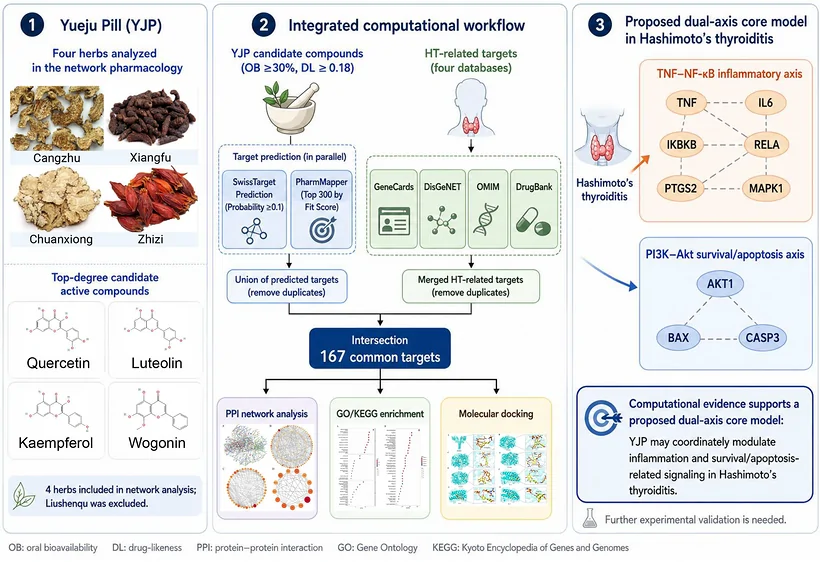

**Abbreviations**
ADCC: antibody‐dependent cell‐mediated cytotoxicity
ADME: absorption, distribution, metabolism, and excretion
BP: biological process
CC: cellular component
CDC: complement‐dependent cytotoxicity
DL: drug‐likeness
GO: Gene Ontology
HT: Hashimoto's thyroiditis
IHC: immunohistochemistry
KEGG: Kyoto Encyclopedia of Genes and Genomes
L‐T4: levothyroxine
MF: molecular function
NF‐κB: nuclear factor kappa‐B
OB: oral bioavailability
PI3K: phosphoinositide 3‐kinase
PPI: protein–protein interaction
ROS: reactive oxygen species
SMILES: simplified molecular‐input line‐entry system
TCM: traditional Chinese medicine
TCMSP: Traditional Chinese Medicine Systems Pharmacology
TgAb: thyroglobulin antibody
TGF‐β: transforming growth factor‐beta
Th17: T helper 17
TNF: tumor necrosis factor
TPOAb: thyroid peroxidase antibody
Treg: T regulatory
YJP: Yueju pill

## 1 Introduction

Hashimoto's thyroiditis (HT) is a common organ‐specific autoimmune disorder characterized pathologically by chronic lymphocytic infiltration and progressive destruction of thyroid follicles [1, 2]. Recent epidemiological studies indicate a global HT prevalence of approximately 7.5% in adults, with a marked gender disparity—affecting women 4 to 10 times more frequently than men [1, 3]. A large‐scale cross sectional survey in China revealed an adult prevalence of HT with positive thyroid antibodies as high as 14.19%, underscoring the substantial disease burden it imposes [4]. As the leading cause of hypothyroidism, the impact of HT extends far beyond thyroid dysfunction. It is associated with multi‐system involvement, profoundly affecting patients' long‐term health and quality of life; for instance, it increases the risks of miscarriage and preterm birth [5], elevates the risk of coronary heart disease by approximately 44% [6], and is associated with increased risks of papillary thyroid carcinoma and primary thyroid lymphoma [7, 8]. Consequently, a deeper understanding of the molecular mechanisms underlying HT progression and the development of therapeutic strategies targeting its pathogenic processes remain important clinical priorities.

At the molecular and cellular level, the core pathology of HT involves a targeted autoimmune attack on thyroid tissue [2]. This process stems from a complex interplay between genetic susceptibility and environmental factors, such as infections and excess iodine [9, 10]. Within this context, thyroid peroxidase (TPO) and thyroglobulin (Tg) released by thyroid follicular cells are recognized by antigen‐presenting cells, which activates B cells to differentiate into plasma cells and produce high titers of TPOAb and TgAb autoantibodies [11, 12]. These antibodies not only serve as diagnostic markers but also contribute to thyroid follicular cell injury through complement‐dependent cytotoxicity (CDC) and antibody‐dependent cell‐mediated cytotoxicity (ADCC). They also recruit inflammatory cells, including macrophages, thereby initiating and perpetuating chronic thyroid inflammation [12, 13].

Central to this process is a profound imbalance in cellular immunity. The immune system in HT patients exhibits significant overactivation and polarization of T helper 1 (Th1) and Th17 cells, coupled with a numerical deficiency and functional exhaustion of regulatory T cells (Tregs) [14, 15]. Th1 cells secrete interferon‐gamma (IFN‐γ), activating macrophages and promoting thyroid follicular cell injury. Th17 cells release pro‐inflammatory cytokines, notably interleukin‐17 (IL‐17), which contributes to amplification of inflammatory responses and tissue damage [16, 17]. In contrast, impaired Treg‐mediated immune suppression through IL‐10 and transforming growth factor‐beta (TGF‐β) further facilitates autoimmune progression [14, 18]. Among these pathological processes, persistent activation of inflammatory signaling pathways, particularly the tumor necrosis factor (TNF)‐nuclear factor kappa‐B (NF‐κB) pathway, plays a central role in cytokine production, inflammatory amplification, and immune‐mediated tissue injury [19, 20]. Meanwhile, the phosphoinositide 3‐kinase (PI3K)‐Akt pathway is critically involved in regulating cellular survival, apoptosis resistance, and tissue homeostasis [21, 22]. Importantly, inflammatory activation and dysregulated apoptosis are closely interconnected during autoimmune tissue destruction [22, 23], suggesting that simultaneous modulation of these two signaling axes may represent a potential therapeutic strategy for HT. Thus, the inflammation–apoptosis interaction constitutes a critical pathological framework underlying progressive thyroid follicular damage and functional impairment in HT.

Current clinical management of HT primarily focuses on restoring thyroid hormone homeostasis, with levothyroxine (L‐T4) replacement therapy remaining the standard treatment for hypothyroidism [24, 25]. However, L‐T4 replacement mainly corrects thyroid hormone deficiency and does not directly target the autoimmune mechanisms responsible for persistent immune dysregulation and thyroid tissue injury. Although thyroid hormone levels can be normalized, some patients continue to experience long‐term symptoms, including fatigue, cognitive impairment, and reduced quality of life [26, 27, 28, 29]. Therefore, complementary therapeutic approaches capable of modulating immune‐inflammatory responses and apoptosis‐related pathways involved in HT progression remain clinically valuable. Given the complex interaction among multiple pathological processes in HT, therapeutic strategies targeting several key molecular nodes may provide advantages over approaches focused on a single pathway.

Against this background, traditional Chinese medicine (TCM), characterized by a “multicomponent, multi‐target, and holistic regulation” strategy, has attracted increasing attention in autoimmune disease management. Recent studies have demonstrated that TCM formulas or natural compounds may regulate immune balance, including Th17/Treg polarization, and suppress inflammatory responses in autoimmune thyroid disease models [30, 31]. However, most previous studies have focused on individual compounds or isolated signaling pathways, whereas the systematic relationships among multiple components, molecular targets, and biological pathways within complex TCM formulas remain insufficiently understood. This limitation restricts the modernization and precise application of TCM‐based therapeutic strategies.

Yueju pill (YJP), derived from Danxi's Mastery of Medicine (Danxi Xinfa), is a classical TCM formula traditionally used for regulating “six stagnations.” Its therapeutic principles, including regulating qi, promoting circulation, and resolving stagnation, are considered relevant to the TCM pathological concept of HT characterized by “qi stagnation, phlegm coagulation, and blood stasis.” Clinical studies involving Yueju‐related preparations have suggested potential benefits in thyroid‐related disorders [32]. However, the molecular basis underlying the potential effects of YJP in HT remains largely unclear. In particular, whether YJP exerts its potential actions through coordinated regulation of multiple components, molecular targets, and signaling networks has not been systematically investigated. A comprehensive pharmacological framework integrating candidate active compounds, HT‐associated targets, and key biological pathways is therefore still lacking.

Network pharmacology and molecular docking provide valuable computational approaches for predicting potential interactions among herbal components, molecular targets, and disease‐associated pathways, particularly for complex TCM formulas with multicomponent characteristics [33, 34]. Compared with conventional single‐target analyses, these approaches enable a systems‐level exploration of potential mechanisms and facilitate the generation of testable mechanistic hypotheses. Therefore, this study integrated network pharmacology and molecular docking approaches to investigate the potential molecular basis of YJP against HT.

Based on the pathological framework of HT involving immune inflammation and apoptosis, we proposed a computationally derived “dual‐axis core” hypothesis, in which YJP may regulate key molecular nodes associated with the TNF–NF‐κB inflammatory axis and the PI3K–Akt survival/apoptosis axis. Specifically, we first identified candidate active components of YJP and predicted their potential targets associated with HT, followed by construction of a herb–component–target–disease network. Subsequently, protein–protein interaction (PPI) network analysis and Gene Ontology (GO)/Kyoto Encyclopedia of Genes and Genomes (KEGG) pathway enrichment analyses were performed to identify potential biological processes and signaling pathways involved. Furthermore, molecular docking was conducted to evaluate the predicted binding potential between representative YJP compounds (quercetin, luteolin, kaempferol, and wogonin) and selected key targets, including PTGS2 (COX‐2), TNF, IKBKB (IKKβ), RELA (p65), CASP3, and AKT1.

This computational study does not provide experimental validation of YJP efficacy or mechanism; rather, it aims to generate a testable dual‐axis regulatory hypothesis and prioritize candidate targets and pathways for future experimental investigation. By integrating systems pharmacology approaches, this work provides computational evidence and a mechanistic framework for further understanding the potential pharmacological basis of YJP in HT.

## 2 Materials and Methods

### 2.1 Network Pharmacology Analysis

#### 2.1.1 Screening of Active Components and Putative Targets of YJP

The chemical constituents of the constituent herbs of YJP, including Atractylodis Rhizoma (Cangzhu), Cyperi Rhizoma (Xiangfu), Chuanxiong Rhizoma (Chuanxiong), and Gardeniae Fructus (Zhizi), were retrieved from the Traditional Chinese Medicine Systems Pharmacology Database and Analysis Platform (TCMSP, https://www.tcmsp‐e.com/). Massa Medicata Fermentata (Liushenqu) was excluded from the present network pharmacology analysis because sufficiently standardized component–target information was not available in the databases used. After removing duplicate and incomplete records, active components were screened according to the TCMSP ADME criteria of oral bioavailability (OB) ≥ 30% and drug‐likeness (DL) ≥ 0.18. Canonical SMILES structures of the selected compounds were obtained from TCMSP and PubChem (https://pubchem.ncbi.nlm.nih.gov/). These SMILES structures were subsequently imported into SwissTargetPrediction (http://www.swisstargetprediction.ch/) and PharmMapper (http://www.lilab‐ecust.cn/pharmmapper/) for potential target prediction. For SwissTargetPrediction, only targets with a predicted probability score ≥ 0.1 were retained. For PharmMapper, the top 300 targets ranked by Fit Score were retained. The predicted targets from the two platforms were then combined using the union operation, redundant targets were removed, and target identifiers were mapped to official gene symbols according to HGNC nomenclature using the UniProt Retrieve/ID mapping tool (https://www.uniprot.org/id‐mapping); ambiguous or obsolete identifiers were manually resolved, yielding the final set of putative targets for YJP. All databases used for compound and target identification were accessed on 17 October 2025.

#### 2.1.2 Screening of HT‐Related Targets

Using “Hashimoto's thyroiditis” as the search term, disease‐associated targets were retrieved from GeneCards (https://www.genecards.org/), OMIM (https://www.omim.org/), DrugBank (https://go.drugbank.com/), and DisGeNET (https://www.disgenet.org/). All databases were accessed on 19 October 2025. Where applicable, targets were restricted to Homo sapiens. To ensure reproducible and database‐specific target inclusion, predefined screening criteria were applied. For GeneCards, only targets with a relevance score ≥ 1 were retained. For DisGeNET, only gene–disease associations with a GDA score (score_gda) > 0 were retained. For OMIM, all gene entries explicitly associated with HT were included, whereas for DrugBank, human protein targets linked to drug entries explicitly associated with HT were retained. The targets obtained from the four databases were merged, duplicate entries were removed, and target identifiers were mapped to official gene symbols according to HGNC nomenclature using the UniProt Retrieve/ID mapping tool; ambiguous or obsolete identifiers were manually resolved, yielding the final set of HT‐related targets for subsequent intersection analysis with YJP‐related targets.

#### 2.1.3 Identification of Common Drug–Disease Targets and PPI Network Construction

The standardized YJP target set was intersected with the HT disease target set to obtain common targets. A Venn diagram was generated for visualization using an online tool (http://bioinformatics.psb.ugent.be/webtools/Venn/). This common target set was then submitted to the STRING database (https://string‐db.org/) to construct a PPI network, with the species set to Homo sapiens and a minimum required interaction score >0.900. Subsequently, Cytoscape software (v3.10.0) (https://cytoscape.org/) was used for network visualization and topological property analysis. Key network topological parameters, including degree, betweenness centrality, and closeness centrality, were calculated. To systematically identify hub targets, a multidimensional integrated strategy was employed. The global median degree of the initial PPI network was 5. Targets with a degree value above this threshold were selected to form a highly connected core target set containing 44 targets. Within this core target set, the local median degree was 10. Targets with a degree value above this secondary threshold were further screened, yielding 13 hub targets. Finally, these 13 hub targets were verified based on their betweenness centrality and closeness centrality values. Functional characterization of common targets was performed through GO and KEGG enrichment analyses. Hub targets within the PPI network were identified based on topological centrality analysis, integrating network connectivity and biological pathway information. Although additional module‐based clustering approaches may provide further insights into network organization, the present study prioritized hub targets through topology‐based screening combined with functional enrichment analysis.

#### 2.1.4 Construction of the “Herb–Component–Target–Disease” Network

Using the CytoHubba plug‐in within Cytoscape software (http://apps.cytoscape.org/apps/cytohubba), the identified hub targets were further evaluated using the degree algorithm. The 13 hub targets were ranked according to their degree values and visualized to illustrate their relative centrality within the PPI network. No additional CytoHubba‐specific cutoff was applied, as the 13 hub targets had already been defined using the progressive median degree screening described in Section 2.1.3. Subsequently, the relationships among YJP, its active components, common drug–disease targets, and HT were integrated to construct a visual “herb–component–target–disease” network. This network was constructed to illustrate the potential multicomponent and multi‐target characteristics of YJP in the regulation of HT‐related biological processes.

#### 2.1.5 GO and KEGG Pathway Enrichment Analysis

To systematically predict the potential biological processes and signaling pathways involved in YJP's treatment of HT, GO and KEGG pathway enrichment analyses were performed on the 167 common drug–disease targets using the R language (R‐4.4.1) (https://www.r‐project.org/). The GO analysis covered three categories: BP, CC, and MF. Enrichment calculations were conducted using R packages such as clusterProfiler (v4.12.6) (https://bioconductor.org/packages/clusterProfiler), with a significance threshold set at an FDR‐adjusted *P*‐value < 0.05. The top 10 most significantly enriched GO terms from each category and the top 30 most significantly enriched KEGG pathways were selected for visualization using R packages like ggplot2 (v3.5.2) (https://cran.r‐project.org/web/packages/ggplot2/). Furthermore, to gain deeper insights into the key pathways, customized schematic diagrams for the TNF and PI3K‐Akt signaling pathways were generated with reference to the KEGG database (https://www.genome.jp/kegg/), clearly annotating the potential targets of YJP intervention in HT on these diagrams.

### 2.2 Molecular Docking Analysis

#### 2.2.1 Preparation of Ligands and Receptors

Based on the herb–component–target network, quercetin, luteolin, kaempferol, and wogonin ranked as the top four components by node degree and were therefore selected as ligands for molecular docking analysis. The three‐dimensional structures of these compounds were obtained from the PubChem database (https://pubchem.ncbi.nlm.nih.gov/) and subjected to energy minimization using Chem3D (v14.0). The energy‐minimized ligand structures were subsequently prepared using AutoDockTools (v1.5.7), including hydrogen‐atom addition, Gasteiger charge assignment, and definition of rotatable bonds, before conversion to PDBQT format for molecular docking analysis.

Nine targets were selected as receptors based on two criteria: (1) hub targets identified from the PPI network analysis and (2) key regulatory nodes involved in the inflammation–apoptosis axis according to KEGG enrichment analysis. Among the 13 PPI hub targets, TNF, IL6, MAPK1, CASP3, and AKT1 were selected because of their central roles in inflammatory regulation, apoptosis regulation, and cell survival signaling. In addition, PTGS2 (COX‐2), IKBKB (IKKβ), RELA, and BAX were selected as important regulatory nodes involved in inflammatory signaling and apoptosis‐related processes and were included among the common drug–disease targets. These nine targets were selected to represent key biological processes involved in inflammatory activation, signal transduction, and apoptosis regulation within the proposed inflammation–apoptosis axis.

The experimentally determined crystal structures of the selected target proteins were obtained from the RCSB Protein Data Bank (https://www.rcsb.org/). Detailed structural information, including protein names, PDB IDs, chain(s) used for docking, resolution, source organism, and co‐crystallized ligands, is provided in Table S1. For RELA, the NF‐κB p65 (RELA)‐containing structure (PDB ID: 3QXY) was selected according to the available crystal structure information.

PyMOL software (version 2.5.0, https://pymol.org/2/) was used for receptor preprocessing. Water molecules were removed, and co‐crystallized ligands were extracted and retained where required for redocking validation or reference–ligand analysis before being removed from the receptor structures used for the main docking analysis. Other heteroatoms were handled according to the structural characteristics of each receptor. AutoDockTools (v1.5.7) was subsequently used for receptor preparation, including hydrogen‐atom addition and Gasteiger charge assignment. The prepared receptor structures were converted into PDBQT format for subsequent molecular docking analysis.

#### 2.2.2 Molecular Docking Process

Molecular docking was performed using AutoDock Vina (v1.2.7) (http://vina.scripps.edu/). The docking grid box was defined according to the co‐crystallized ligand‐binding site when an appropriate small‐molecule ligand was available. For targets without a suitable co‐crystallized small‐molecule ligand, the grid box was centered on the reported ligand‐binding region according to available structural information. The grid center coordinates and dimensions were determined from the docking configuration files for each target–compound docking system. Detailed grid‐box parameters, including center coordinates (*x*, *y*, and *z*), grid size, and the basis used for grid definition, are provided in Table S2.

For each docking system, the pose with the lowest predicted binding energy was selected as the representative binding mode. Docking scores calculated by AutoDock Vina were used as comparative computational indicators of predicted ligand–protein interactions. More negative docking scores were interpreted as relatively more favorable predicted interactions within a given target system; however, these scores were not considered direct measurements of experimental binding affinity. Representative docking conformations were visualized using PyMOL.

To evaluate the reproducibility of the docking protocol, native‐ligand redocking was performed for receptors containing suitable co‐crystallized small‐molecule ligands. The corresponding ligands were extracted from the experimental crystal structures and re‐docked into their original binding sites using the same AutoDock Vina protocol as that applied in the main docking analysis. Heavy‐atom root‐mean‐square deviation (RMSD) values were calculated between the experimentally determined crystal poses and the best‐scored redocked poses, with RMSD values <2.0 Å considered indicative of successful reproduction of the experimental binding mode. For MAPK1, five independent redocking runs with different random seeds were additionally performed because of the conformational flexibility of the co‐crystallized inhibitor SCH772984. For the 3QXY complex, *S*‐adenosylmethionine (SAM) was additionally redocked at its cofactor‐binding site as a site‐specific structural check because the RELA component is represented by a peptide fragment rather than a redockable small‐molecule ligand. The complete redocking results are provided in Table S3.

For comparative docking evaluation, co‐crystallized ligands were used as structural reference ligands where appropriate. For targets without a suitable redockable small‐molecule co‐crystallized ligand, reported small‐molecule inhibitors, antagonists, or binding ligands were used as reference compounds. All reference compounds were docked using the same AutoDock Vina protocol and the corresponding target‐specific grid settings used for the YJP‐derived compounds. reference–ligand docking scores were interpreted only as within‐target comparative values and not as absolute measurements of experimental binding affinity. For the 3QXY complex, SAM was considered only as a site‐specific structural reference at the SETD6 cofactor‐binding site and not as a direct positive‐control ligand for RELA. The reference–ligand docking results are summarized in Table S4.

Key intermolecular interactions were further characterized from the representative lowest‐energy docking poses using predefined geometric criteria. Hydrogen‐bond contacts were identified between ligand and receptor N/O/S atoms within a heavy‐atom distance of 3.5 Å. Hydrophobic contacts were defined using a 4.5 Å cutoff between ligand carbon atoms and side‐chain carbon atoms of hydrophobic residues. Potential π–π interactions were identified when the centroid distance between a ligand aromatic ring and an aromatic residue (Phe, Tyr, Trp, or His) was within 5.5 Å, whereas potential salt bridges were identified between oppositely charged ligand and receptor groups within 4.5 Å. Interaction geometry was analyzed using RDKit (v2025.09.4) based on the AutoDock Vina/PyMOL‐derived docking poses. Detailed ligand–residue interactions are provided in Table S5A. For receptors containing suitable co‐crystallized structural reference ligands, residues contacted by the YJP‐derived compounds were further compared with those contacted by the corresponding reference ligands (Table S5B). For the 3QXY complex, comparison with SAM was interpreted only as a site‐specific structural reference rather than as evidence of direct RELA–ligand binding.

## 3 Results

### 3.1 Network Pharmacology Results

#### 3.1.1 Active Components and Targets of YJP

Initially, 440 chemical constituents were retrieved from the TCMSP database for the four analyzed herbs of YJP, including 49 compounds from Atractylodis Rhizoma (Cangzhu), 104 compounds from Cyperi Rhizoma (Xiangfu), 189 compounds from Chuanxiong Rhizoma (Chuanxiong), and 98 compounds from Gardeniae Fructus (Zhizi). After removal of duplicate and incomplete records, candidate active compounds were screened according to the TCMSP ADME criteria of OB ≥30% and DL ≥0.18. This process resulted in 32 candidate active compounds, including 4 derived from Atractylodis Rhizoma, 6 from Chuanxiong Rhizoma, 16 from Cyperi Rhizoma, and 6 from Gardeniae Fructus (Table 1).

**TABLE 1:** Candidate active components of YJP filtered by ADME criteria.

Molecule ID	Components	Sources
MOL000173	Wogonin	Cangzhu
MOL000184	NSC63551	Cangzhu
MOL000188	3β‐Acetoxyatractylone	Cangzhu
MOL000085	β‐Daucosterol_qt	Cangzhu
MOL001494	Mandenol	Chuanxiong
MOL002135	Myricanone	Chuanxiong
MOL002140	Perlolyrine	Chuanxiong
MOL002157	Wallichilide	Chuanxiong
MOL000359	Sitosterol	Chuanxiong
MOL000433	FA	Chuanxiong
MOL003044	Chryseriol	Xiangfu
MOL000354	Isorhamnetin	Xiangfu
MOL003542	8‐Isopentenyl‐kaempferol	Xiangfu
MOL000358	β‐Sitosterol	Xiangfu
MOL004053	Isodalbergin	Xiangfu
MOL004058	Khell	Xiangfu
MOL010489	Resivit	Xiangfu
MOL004068	Rosenonolactone	Xiangfu
MOL004071	Hyndarin	Xiangfu
MOL004074	Stigmasterol glucoside_qt	Xiangfu
MOL004077	Sugeonyl acetate	Xiangfu
MOL000422	Kaempferol	Xiangfu
MOL000449	Stigmasterol	Xiangfu
MOL000006	Luteolin	Xiangfu
MOL000098	Quercetin	Xiangfu
MOL001406	Crocetin	Zhizi
MOL001941	Ammidin	Zhizi
MOL004561	Sudan III	Zhizi
MOL001942	Isoimperatorin	Zhizi
MOL002883	Ethyl oleate (NF)	Zhizi
MOL003095	5‐Hydroxy‐7‐methoxy‐2‐(3,4,5‐trimethoxyphenyl)chromone	Zhizi
MOL007245	3‐Methylkempferol	Zhizi

#### 3.1.2 Screening of Disease Targets

Using “HT” as the search term, disease‐related targets were collected from GeneCards, OMIM, DrugBank, and DisGeNET. After merging and removing duplicates, 4608 potential HT targets were obtained.

#### 3.1.3 Common Targets and Network Construction

The intersection of drug and disease targets yielded 167 common targets (Figure 1A). To visualize YJP's “multicomponent, multi‐target” action characteristics, a “drug–components–targets–disease” network was constructed using Cytoscape (Figure 1B). This network illustrated the potential relationships between the 32 candidate active compounds and the 167 common targets. Based on node degree in the network, candidate active compounds with high connectivity—quercetin, luteolin, kaempferol, and wogonin—were identified as network hubs and selected as ligands for subsequent molecular docking.

**FIGURE 1:**
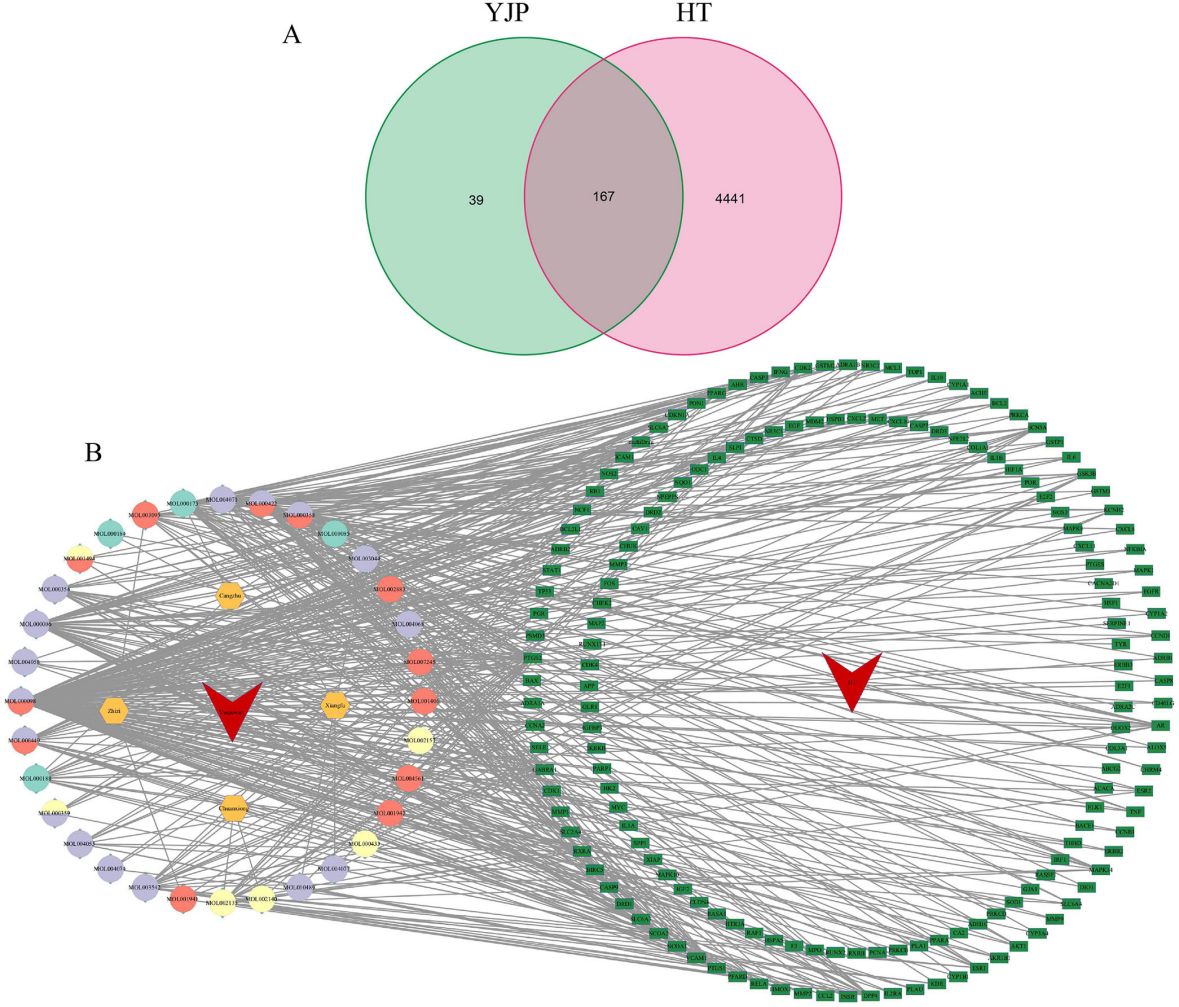
Network of “herb‐components‐targets‐disease” and common targets for YJP in the treatment of Hashimoto's thyroiditis. (A) Venn diagram of common drug–disease targets. (B) Interaction network of YJP, its active components, common targets, and Hashimoto's thyroiditis.

#### 3.1.4 PPI Network and Core Target Screening

To systematically reveal the core protein interaction modules of YJP intervention in HT, the 167 common targets were imported into the STRING database to construct an initial PPI network with high confidence (> 0.900). After filtering out isolated targets, a network of 146 targets was obtained (Figure 2A). Cytoscape was used for visualization, with node size and color depth representing degree (higher degree = larger/darker nodes) (Figure 2B). To identify highly connected nodes, a progressive screening strategy based on median degree was adopted: first, targets with degree above the global median were selected to form a high‐connectivity cluster (44 targets), representing a tightly interconnected core subnetwork (Figure 2C). Second, within this cluster, targets with degree above the local median were further screened, yielding 13 core targets: FOS, MYC, CCND1, MAPK1, AKT1, IL6, ESR1, TNF, CASP3, CDKN1A, BCL2, TP53, and MDM2 (Figure 2D). These 13 targets, exhibiting high centrality in both global and core networks, were defined as hub targets. To further evaluate the potential involvement of YJP in the “inflammation–apoptosis” axis, key targets were selected for molecular docking. Among the 13 PPI hub targets, TNF, IL6, MAPK1, CASP3, and AKT1 were selected because of their relevance to inflammatory regulation, apoptosis, and cell‐survival signaling. In addition, PTGS2 (COX‐2), IKBKB (IKKβ), RELA (p65), and BAX were selected from the 167 common targets as functionally relevant nodes associated with inflammatory and apoptosis‐related processes. These 9 key targets represent major processes involving inflammatory signaling, signal transduction, and apoptosis regulation, providing a basis for molecular‐level assessment of the proposed “dual‐axis core” model.

**FIGURE 2:**
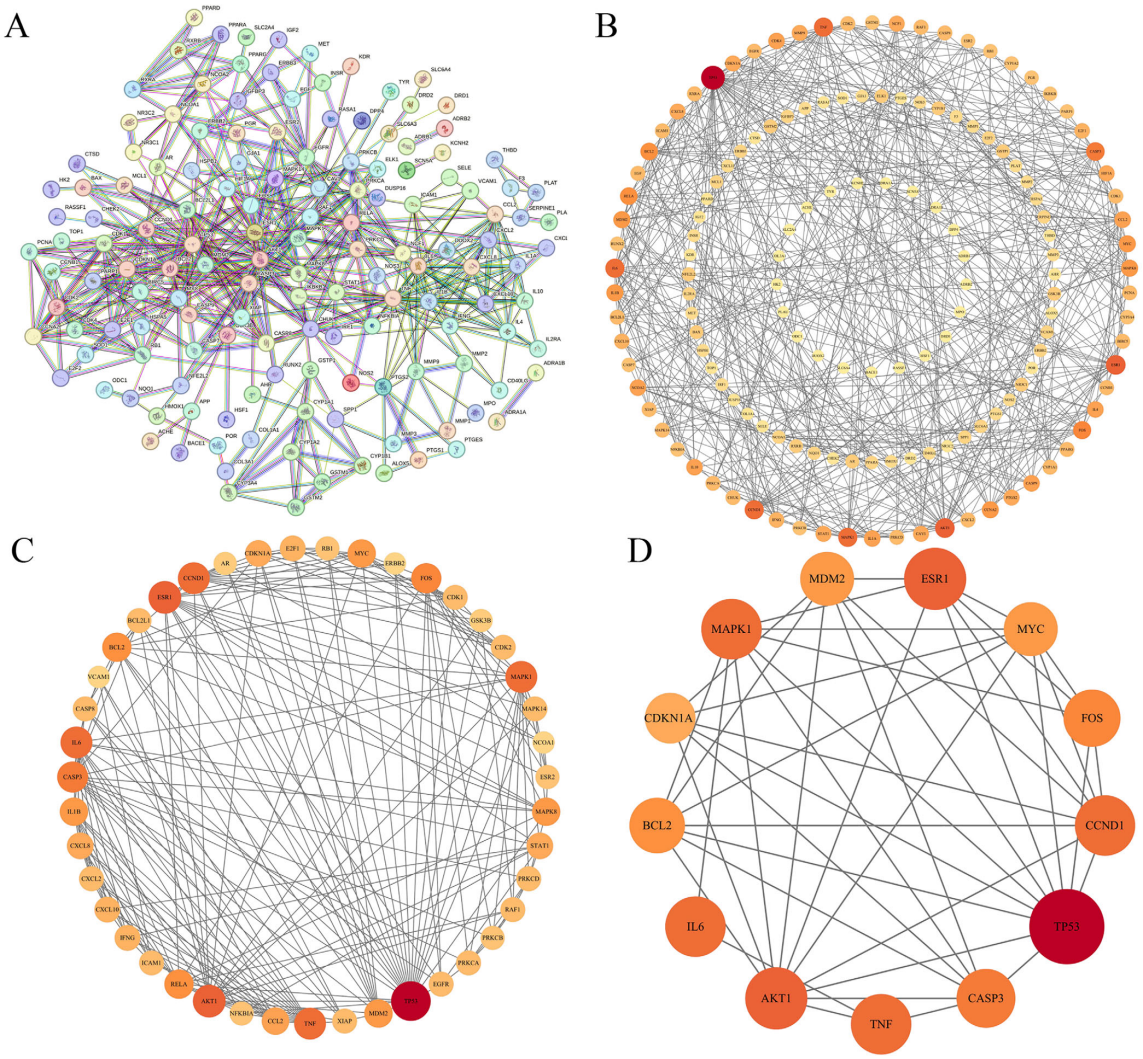
PPI network and progressive screening of core targets for YJP intervention in Hashimoto's thyroiditis. (A) Initial PPI network constructed using the STRING database. (B) Overall PPI network visualized with Cytoscape (node size and color depth represent the degree value). (C) High‐connectivity target subnetwork after preliminary screening based on the median degree value. (D) Final core targets (*n* = 13) identified using the progressive median strategy.

#### 3.1.5 GO and KEGG Enrichment Analysis

GO enrichment analysis (Figure 3A) showed that common targets were significantly enriched in biological processes such as response to molecule of bacterial origin, response to reactive oxygen species, and cellular response to chemical stress; cellular components including membrane rafts and transcription regulator complexes; and molecular functions such as transcription factor binding and ubiquitin‐like protein ligase binding. KEGG pathway analysis (Figure 3B) indicated significant enrichment in pathways including the PI3K‐Akt signaling pathway, TNF signaling pathway, IL‐17 signaling pathway, Th17 cell differentiation, and apoptosis, providing pathway‐level support for the “dual‐axis core” hypothesis. To systematically evaluate this hypothesis at the pathway level, in‐depth visualization of key pathways was performed. In the TNF signaling pathway, multiple core YJP targets (e.g., TNF, CASP3, IL6, and MAPK1) were distributed across multiple key steps associated with membrane receptor signaling, NF‐κB transcriptional regulation, and apoptosis‐related processes (Figure 4A), illustrating the potential involvement of these targets in the proposed “inflammation–apoptosis” axis. Importantly, in the PI3K‐Akt signaling pathway, multiple core targets (e.g., AKT1, IL6, MYC, and CCND1) were densely distributed at key nodes (Figure 4B). As a central pathway regulating cell survival, proliferation, and metabolism, its coordinated involvement with the TNF pathway suggests that YJP may modulate TNF‐related inflammatory signaling together with PI3K‐Akt‐related survival signaling, supporting a potential coordinated dual‐axis regulatory pattern.

**FIGURE 3:**
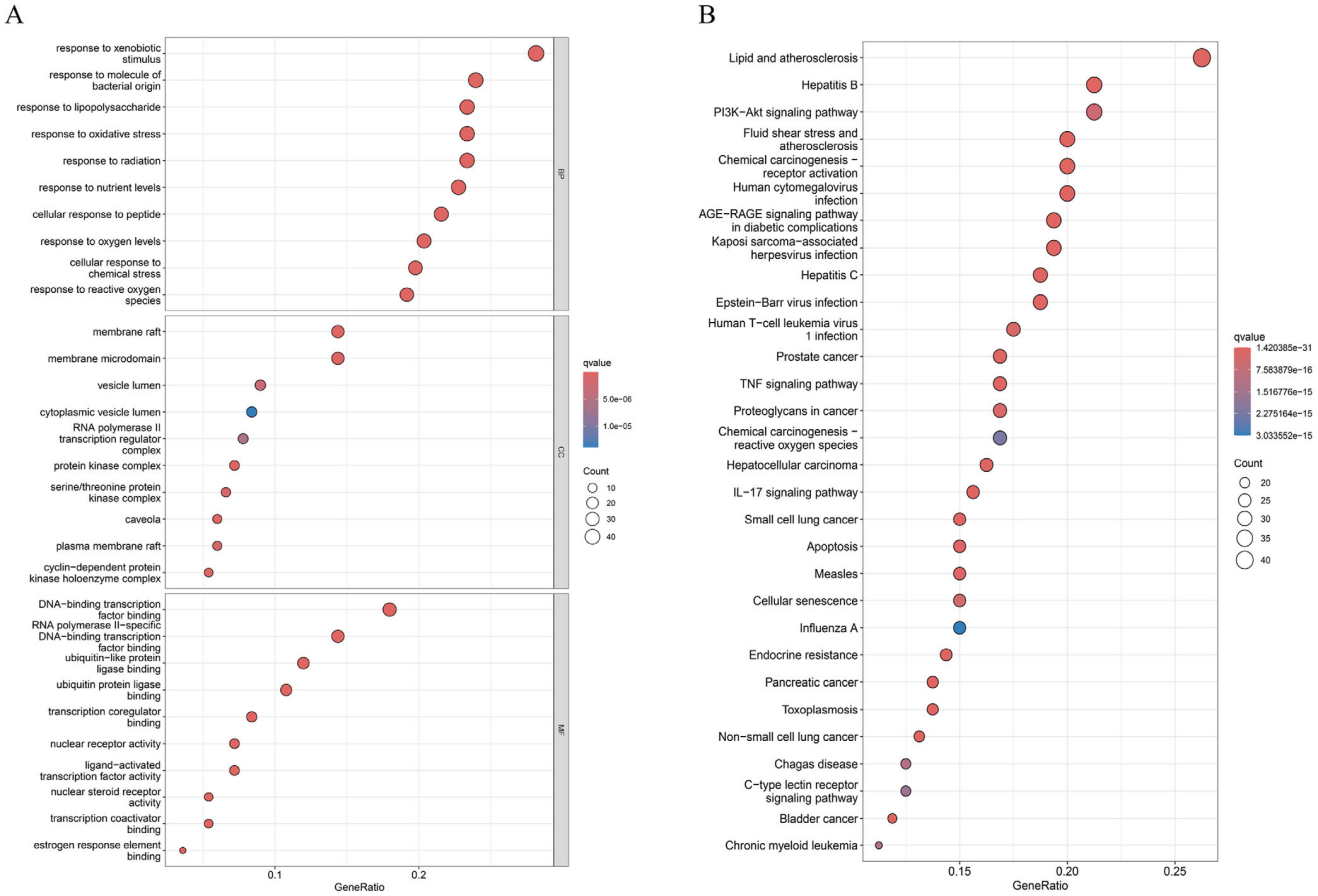
GO and KEGG pathway enrichment analysis of common targets for YJP in treating Hashimoto's thyroiditis. (A) Bar graph of GO enrichment analysis (displaying the top 10 significantly enriched terms for biological process, cellular component, and molecular function). (B) Bubble chart of KEGG pathway enrichment analysis (displaying the top 30 significantly enriched pathways). The results suggest that YJP significantly acts on key pathways such as TNF and PI3K‐Akt.

**FIGURE 4:**
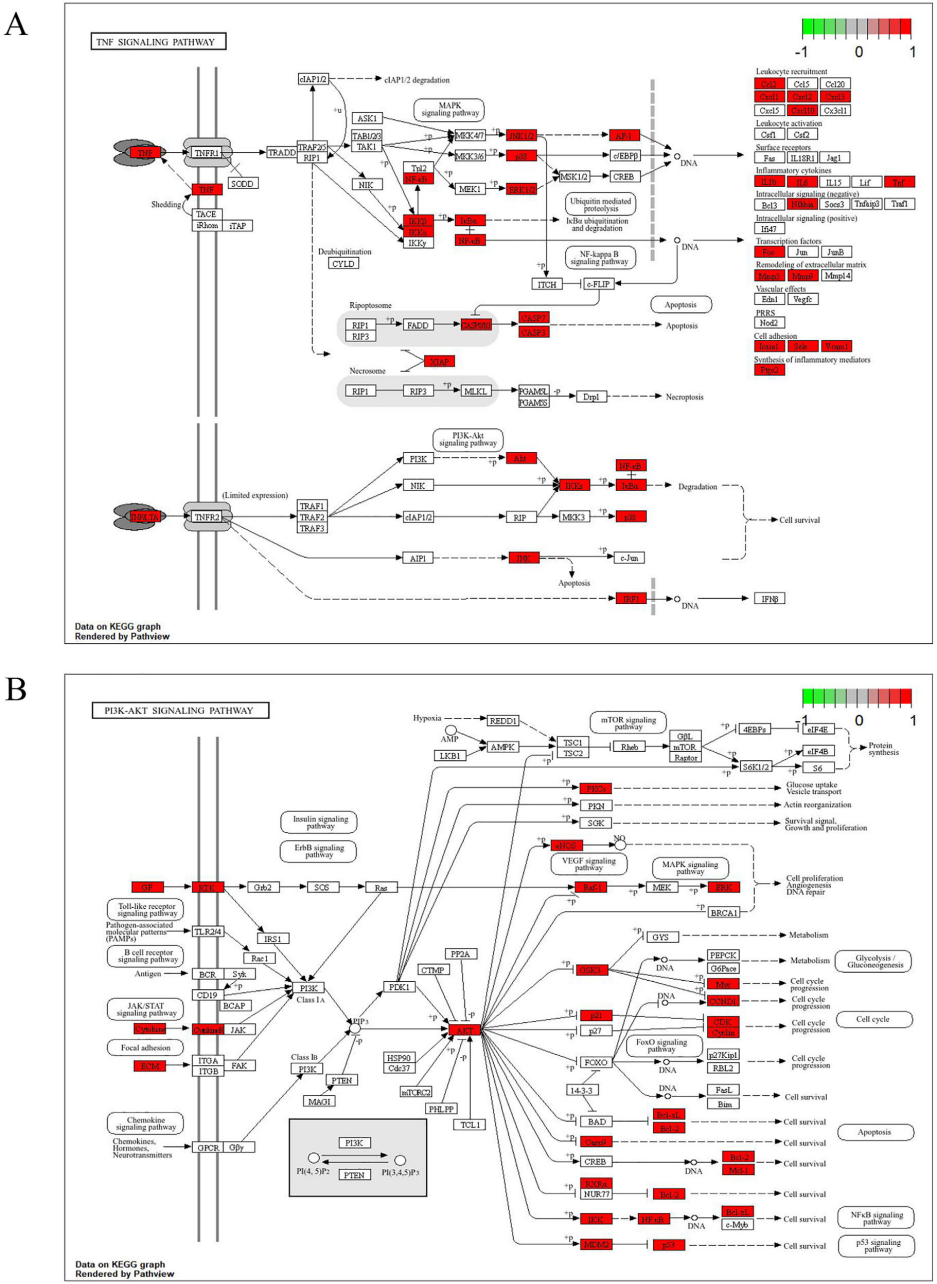
Localization of core targets of YJP in key pathways of the “dual‐axis core” model. (A) TNF signaling pathway map, with potential targets of YJP intervention annotated. The targets cover key nodes from inflammation initiation to apoptosis execution. (B) PI3K‐Akt signaling pathway map, with potential targets of YJP intervention annotated. The targets are densely distributed at core hubs regulating cell survival and apoptosis.

### 3.2 Molecular Docking Results

To evaluate the potential interactions between YJP‐derived active compounds and core targets involved in the proposed “inflammation–apoptosis” dual‐axis model, molecular docking analysis was performed using key targets identified by network pharmacology. The predicted binding energies varied among different ligand–target pairs, and relatively more negative docking scores were observed for several compound–target combinations, suggesting potentially favorable predicted interactions (Table 2). Representative 3D docking conformations are presented in Figures 5, 6, 7, 8.

**TABLE 2:** Molecular docking binding energies (kcal/mol) of core active components of YJP with Key targets on the “inflammation–apoptosis” axis.

Target (PDB ID)	Quercetin	Luteolin	Kaempferol	Wogonin
TNF (1TNF)	−9.18	−9.23	−9.20	−8.65
RELA (3QXY)	−9.17	−9.01	−9.20	−9.01
IKBKB (4KIK)	—	—	−8.20	—
PTGS2 (5F19)	−8.33	−9.02	−8.87	−8.88
IL6 (1ALU)	−6.37	−7.12	—	−6.39
MAPK1 (4QTA)	−9.47	−9.68	—	—
AKT1 (1H10)	−6.17	−6.26	−6.25	−6.12
BAX (4BD6)	−6.40	—	−7.02	−6.37
CASP3 (1GFW)	−6.91	−7.81	−7.26	−7.61

**FIGURE 5:**
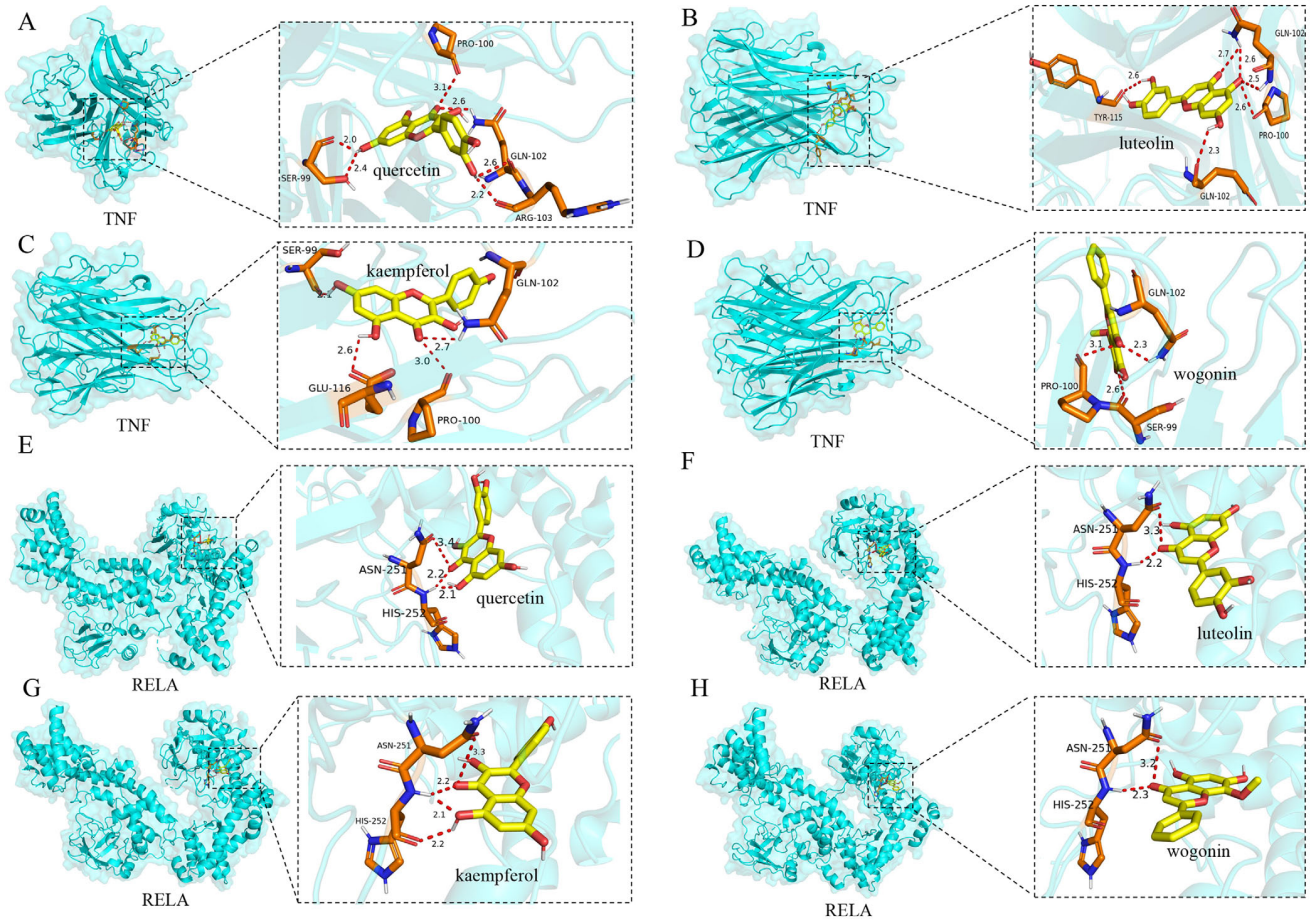
Molecular docking of TNF and RELA with herbal ligands. (A) TNF with quercetin. (B) TNF with luteolin. (C) TNF with kaempferol. (D) TNF with wogonin. (E) RELA with quercetin. (F) RELA with luteolin. (G) RELA with kaempferol. (H) RELA with wogonin.

**FIGURE 6:**
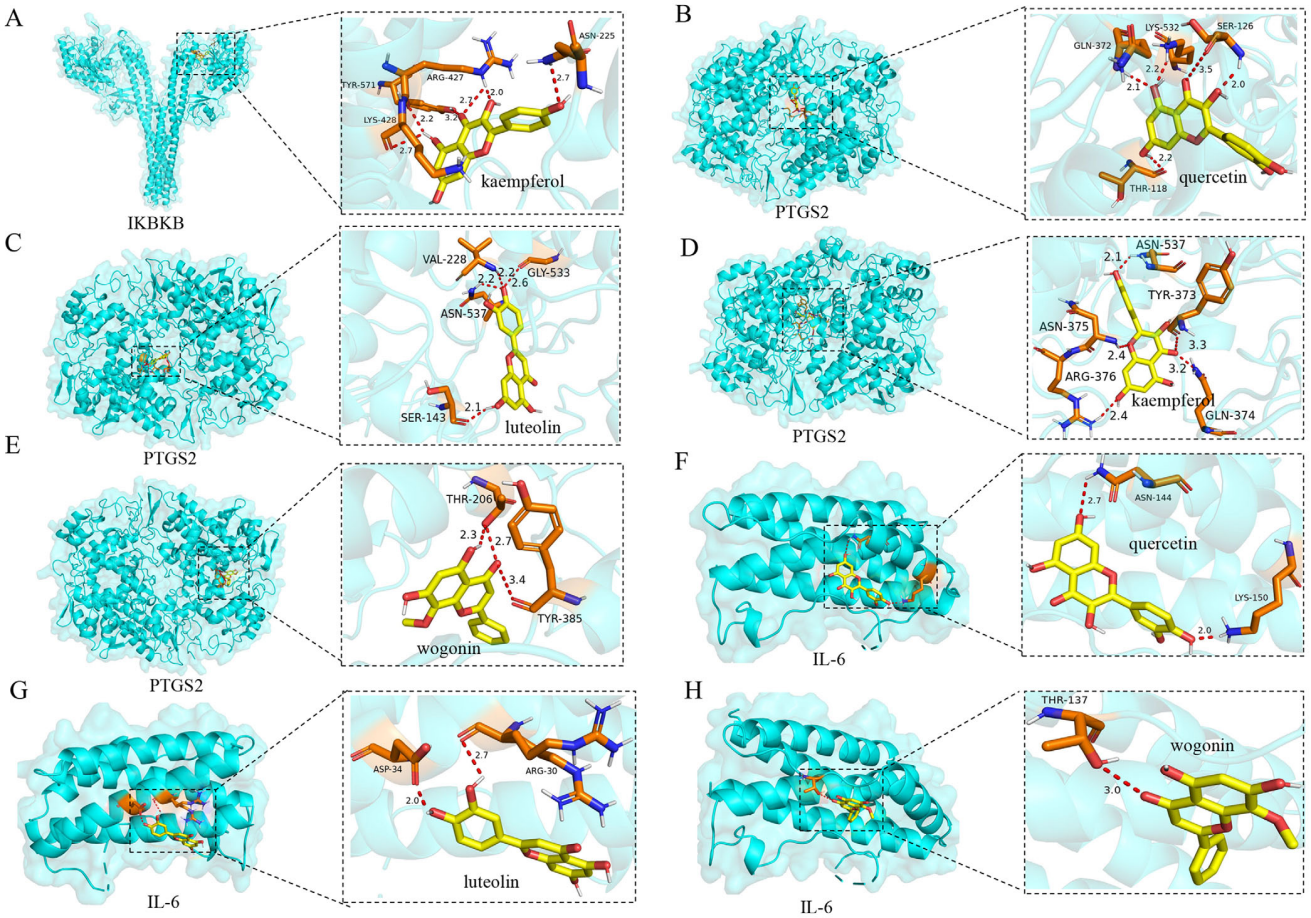
Molecular docking of IKBKB, PTGS2, and IL‐6 with herbal ligands. (A) IKBKB with kaempferol. (B) PTGS2 with quercetin. (C) PTGS2 with luteolin. (D) PTGS2 with kaempferol. (E) PTGS2 with wogonin. (F) IL6 with quercetin. (G) IL6 with luteolin. (H) IL6 with wogonin.

**FIGURE 7:**
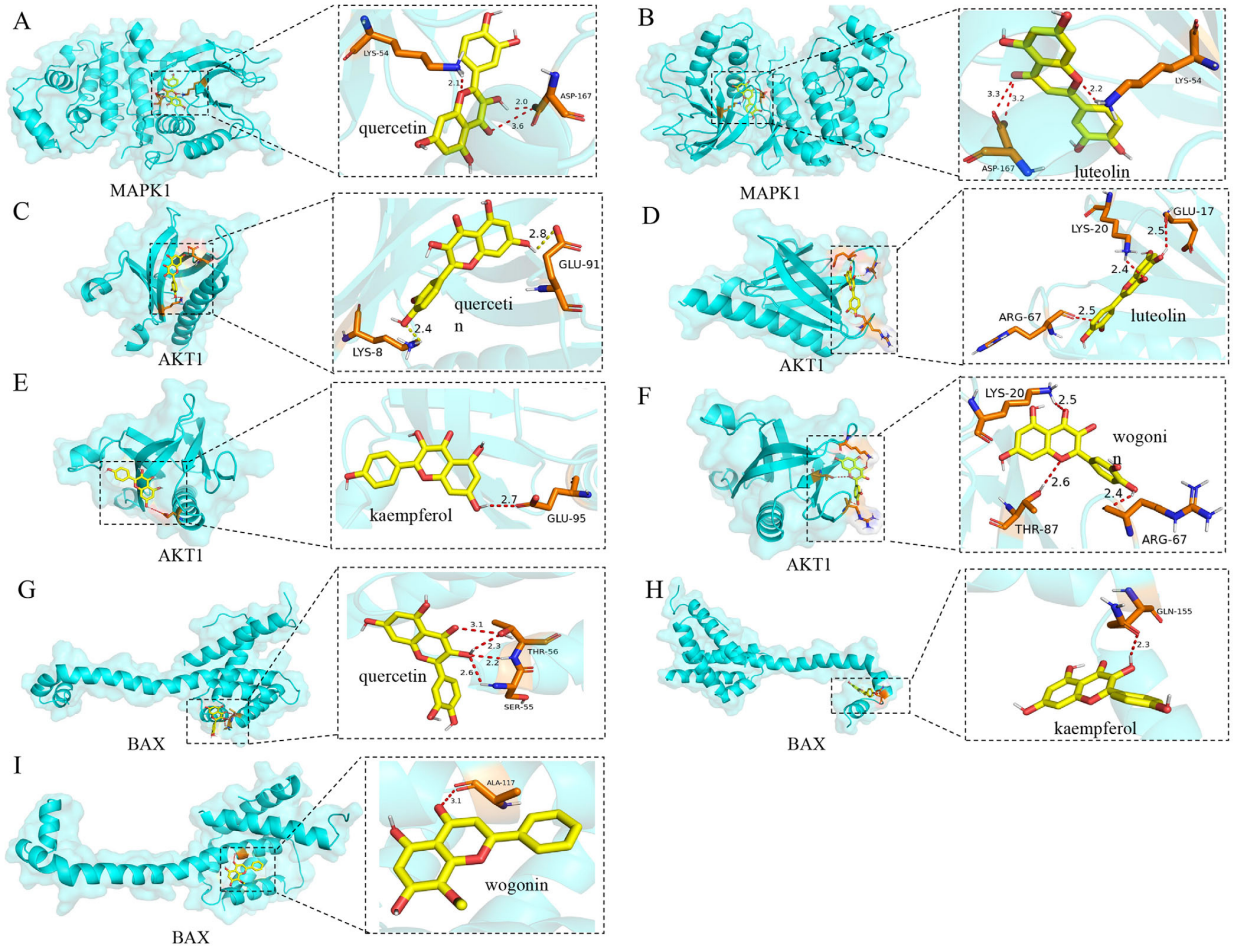
Molecular docking of MAPK1, AKT1, and BAX with herbal ligands. (A) MAPK1 with quercetin. (B) MAPK1 with luteolin. (C) AKT1 with quercetin. (D) AKT1 with luteolin. (E) AKT1 with kaempferol. (F) AKT1 with wogonin. (G) BAX with quercetin. (H) BAX with kaempferol. (I) BAX with wogonin.

**FIGURE 8:**
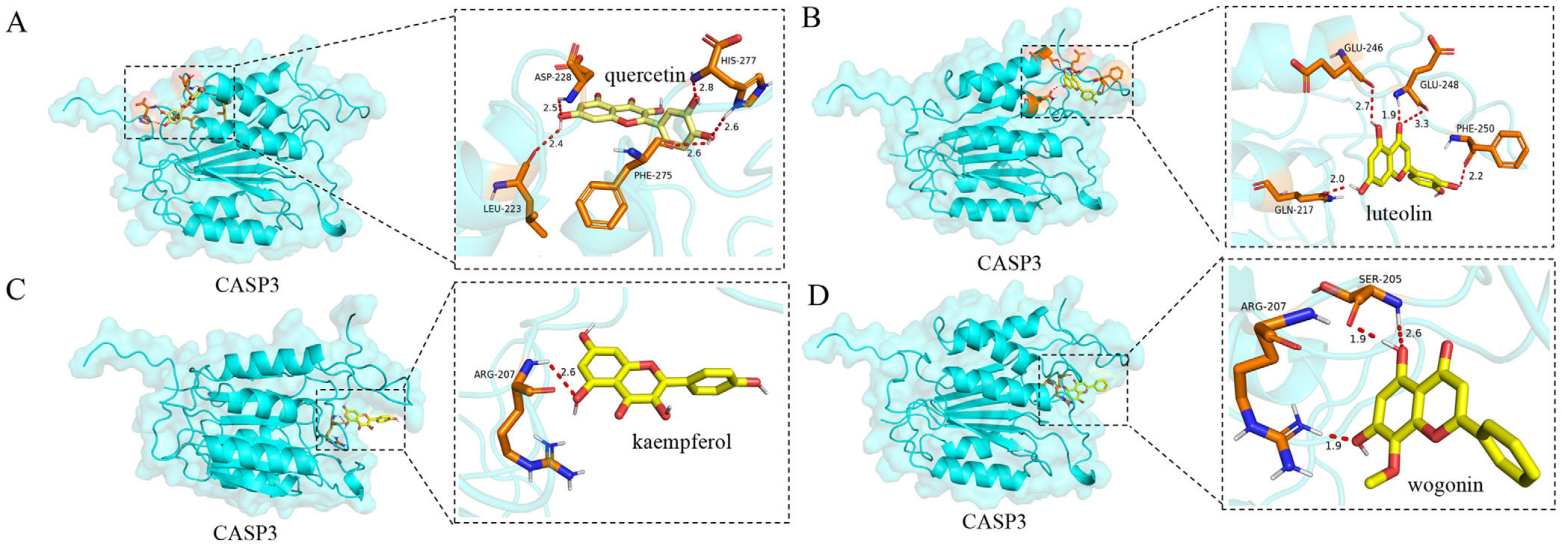
Molecular docking of CASP3 with herbal ligands. (A) CASP3 with quercetin. (B) CASP3 with luteolin. (C) CASP3 with kaempferol. (D) CASP3 with wogonin.

Within the TNF–NF‐κB inflammatory axis, luteolin, quercetin, and kaempferol exhibited predicted binding energies of −9.23, −9.18, and −9.20 kcal/mol toward TNF, respectively. Kaempferol showed a predicted binding energy of −8.20 kcal/mol toward IKBKB (IKKβ). The four major compounds also showed relatively negative predicted docking scores in the 3QXY system. In addition, the selected compounds exhibited predicted interactions with other inflammation‐associated targets, including PTGS2 (COX‐2), IL6, and MAPK1, with docking scores ranging from −6.37 to −9.68 kcal/mol depending on the specific compound–target combination. These values were interpreted comparatively rather than according to predefined absolute affinity thresholds.

Within the PI3K–Akt survival/apoptosis axis, luteolin and wogonin exhibited predicted binding energies of −7.81 and −7.61 kcal/mol toward CASP3, respectively, while kaempferol showed a predicted binding energy of −7.02 kcal/mol toward BAX. All four compounds were also predicted to interact with AKT1, with docking scores ranging from −6.12 to −6.26 kcal/mol.

Redocking analysis was performed to assess the reproducibility of the docking protocol. CASP3, IKBKB, MAPK1, and AKT1 yielded heavy‐atom RMSD values of 1.94, 0.49, 0.16, and 0.48 Å, respectively, all below the predefined 2.0 Å criterion (Table S3). For MAPK1, the reported value of 0.16 Å represented the best‐scored pose obtained from five independent redocking runs. In the 3QXY complex, redocking of the co‐crystallized SETD6 cofactor SAM at its own cofactor‐binding site yielded an RMSD of 2.45 Å and was therefore treated as an additional site‐specific structural check of the docking setup rather than as a direct assessment of ligand binding to RELA. Native‐ligand redocking was not applicable to TNF, IL6, PTGS2, and BAX because suitable small‐molecule co‐crystallized ligands were unavailable.

Reference–ligand docking provided an additional within‐target comparison (Table S4). For AKT1, the four YJP‐derived compounds yielded docking scores of −6.12 to −6.26 kcal/mol, compared with −6.55 kcal/mol for the co‐crystallized 4IP reference ligand. For TNF, the YJP compounds yielded scores of −8.65 to −9.23 kcal/mol compared with −9.88 kcal/mol for the reference inhibitor SPD304, whereas for PTGS2 the YJP compounds yielded scores of −8.33 to −9.02 kcal/mol compared with −9.18 kcal/mol for celecoxib. Conversely, YJP‐derived compounds showed less negative docking scores than the corresponding co‐crystallized reference ligands for CASP3, IKBKB, and MAPK1. These comparisons were interpreted only within individual target systems and were not considered evidence of equivalent or superior experimental binding affinity. Because SAM in the 3QXY structure binds to the SETD6 cofactor site rather than serving as a direct RELA ligand, its docking score was used only as a site‐specific structural reference.

Analysis of representative docking poses identified hydrogen‐bond, hydrophobic, and π–π contacts involving residues within the predicted binding regions (Table S5A). MAPK1–luteolin formed hydrogen‐bond contacts with LYS54A and ASP167A, hydrophobic contacts involving TYR36A, ILE56A, ILE103A, and VAL39A, and a π–π interaction with TYR36A. CASP3–luteolin formed polar contacts involving GLU248B, PHE250B, GLU246B, ASP211B, and GLN217B, together with hydrophobic and aromatic contacts involving PHE247B, TRP214B, PHE250B, and TRP206B. For receptors with suitable co‐crystallized reference ligands, partial overlap was observed between residues contacted by YJP‐derived compounds and those contacted by the corresponding experimentally determined reference ligands (Table S5B), providing additional structural support for the plausibility of the predicted poses. Representative two‐dimensional interaction diagrams for CASP3–luteolin, MAPK1–luteolin, TNF–quercetin, and PTGS2–luteolin are shown in Figure 9.

**FIGURE 9:**
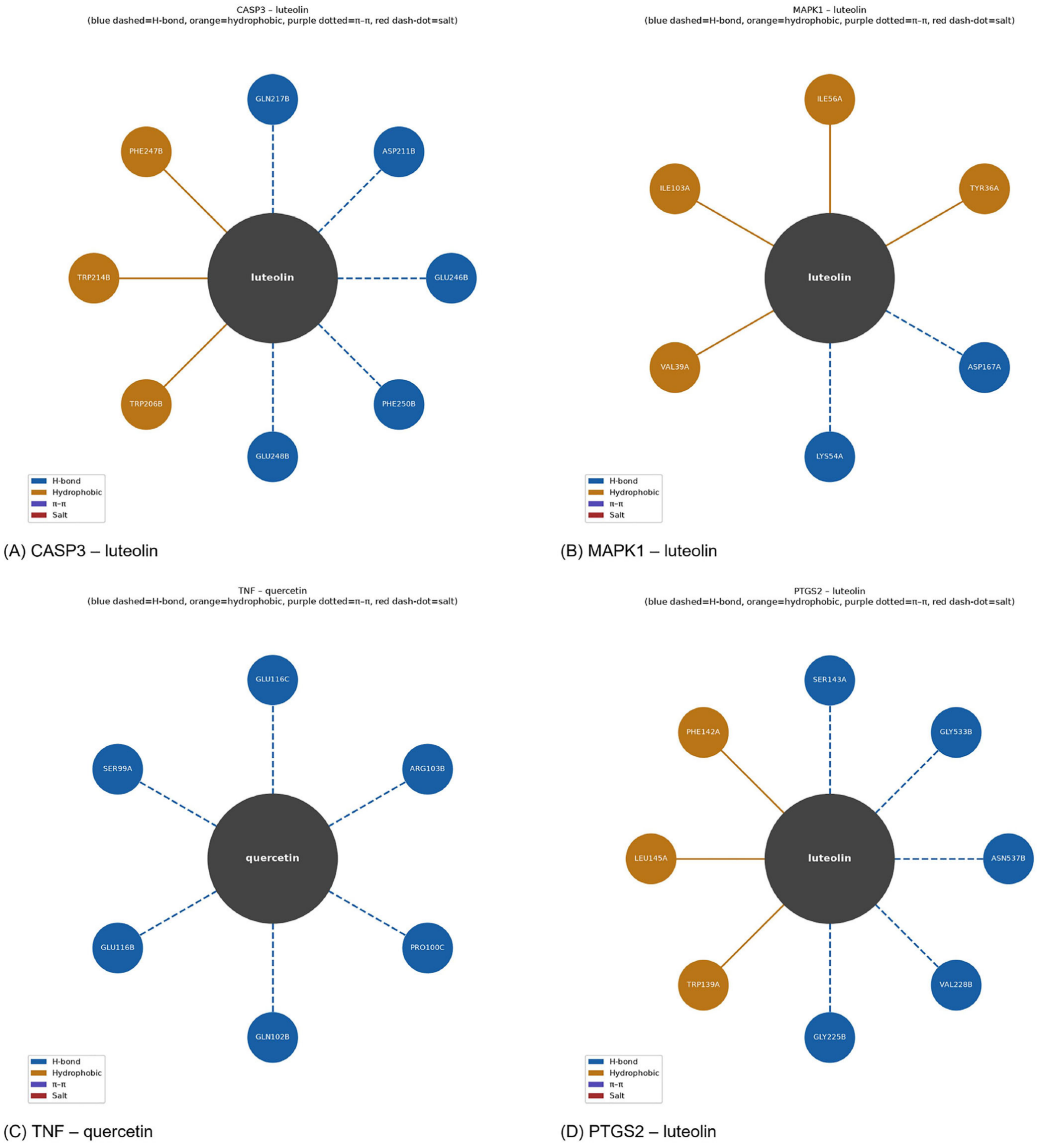
Representative 2D interaction diagrams of YJP‐derived active compounds with key target proteins. (A) CASP3–luteolin. (B) MAPK1–luteolin. (C) TNF–quercetin. (D) PTGS2–luteolin. The diagrams illustrate the major ligand–residue interactions identified from the representative docking poses, including hydrogen bonds and hydrophobic contacts. Detailed interaction profiles for all docked compound–target pairs, including π–π interactions and salt bridges where identified, are provided in Table S5A. Comparisons with residues contacted by the corresponding structural reference ligands are provided in Table S5B.

Overall, molecular docking analysis suggested that the major active compounds of YJP may potentially interact with multiple targets associated with inflammatory regulation, signal transduction, and apoptosis‐related processes within the proposed “inflammation–apoptosis” dual‐axis model. The redocking analysis, reference–ligand comparison, and residue‐level interaction analysis provide complementary computational support for the predicted docking modes; however, these findings remain computational predictions and require biochemical and experimental validation to establish actual molecular binding and functional effects.

## 4 Discussion

This study integrates network pharmacology and molecular docking to predict the multicomponent, multi‐target mechanism of YJP in treating HT. Our findings support a therapeutic potential arising from the coordinated intervention of two core signaling pathways in HT pathology: the “TNF–NF‐κB‐driven inflammatory axis” and the “PI3K–Akt‐regulated cell survival/apoptosis pathway”. Network pharmacology revealed a significant enrichment of YJP's core targets within these pathways, while molecular docking predicted favorable binding interactions between its active components and these targets. These results collectively provide computational support for a novel “dual‐axis core” model, providing a new perspective for understanding YJP's systemic mechanism of action against HT.

### 4.1 The “Dual‐Axis Core”: Parallel Intervention on Inflammation and Apoptosis

The pathology of HT is characterized by aberrant autoimmune inflammation and progressive apoptosis of thyroid follicular cells. NF‐κB pathway overactivation is an established driver of local thyroid inflammation, promoting lymphocyte infiltration and follicular destruction through upregulation of cytokines like TNF‐α and IL‐6 [35, 36, 37]. Concurrently, dysregulated apoptosis, particularly via the Fas/FasL‐mediated death receptor pathway, is a direct cause of thyrocyte loss [12]. Current therapies often target single pathways, lacking a holistic strategy for the “inflammation–apoptosis” network. Our network pharmacology analysis suggests that YJP may actively and simultaneously modulate these two core pathways. Its targets are not randomly distributed but are significantly enriched within a core “inflammation–apoptosis” regulatory axis involving TNF, IL6, RELA, CASP3, and AKT1. This specific enrichment pattern highlights YJP's multi‐target nature and provides computational evidence for its coordinated involvement in key pathological processes of HT, supporting the “dual‐axis core” hypothesis.

#### 4.1.1 Multi‐Level Intervention on the TNF–NF‐κB Axis

Our findings indicate that YJP may employ a multicomponent strategy to modulate the TNF–NF‐κB axis at multiple levels of the signaling cascade. At the stage of signal initiation, molecular docking revealed that quercetin, luteolin, and kaempferol exhibited relatively favorable predicted binding to TNF (−9.18, −9.23, and −9.20 kcal/mol, respectively), supporting the potential involvement of these compounds in TNF‐related inflammatory regulation. Moving to intracellular signal transduction, kaempferol showed a relatively favorable predicted binding energy for IKBKB (IKKβ) (−8.20 kcal/mol), indicating its potential interaction with this key regulatory kinase [38, 39]. At the level of NF‐κB‐related transcriptional regulation, all four core components showed relatively favorable docking scores in the 3QXY SETD6–RELA peptide complex (≤ −9.0 kcal/mol). Given the structural characteristics of 3QXY, these results were interpreted as site‐specific computational evidence associated with the SETD6–RELA system rather than as direct evidence of ligand binding to RELA, while remaining consistent with the proposed coordinated modulation of NF‐κB‐related signaling [40].

Furthermore, YJP's potential influence extends beyond the core NF‐κB cascade to key associated nodes, forming a multi‐target inflammatory regulatory network. The relatively favorable predicted binding of luteolin and quercetin to MAPK1 (−9.68 and −9.47 kcal/mol) supports their potential involvement in modulation of complementary inflammatory signaling pathways. Additionally, predicted interactions with IL6 and PTGS2 (COX‐2, a classic NSAID target [41]), with PTGS2 docking energies reaching −9.02 kcal/mol, further highlight the multi‐target characteristics of YJP. Collectively, these findings provide computational support for the potential coordinated regulation of multiple nodes spanning inflammatory signal initiation, intracellular signal transduction, and downstream transcriptional regulation.

#### 4.1.2 Modulating Apoptosis via the PI3K‐Akt Pathway

Our integrated analysis further supports the anti‐apoptotic potential of YJP, which is closely linked to the significantly enriched PI3K‐Akt pathway. This potential regulatory effect may involve multiple tiers of the apoptotic cascade. At the level of apoptosis execution, luteolin, wogonin, quercetin, and kaempferol showed predicted interactions with CASP3 (with binding energies of −7.81, −7.61, −6.91, and −7.26 kcal/mol, respectively). Given the documented elevation of CASP3 activity in HT thyroid tissue [42], these predicted interactions suggest that YJP components may influence CASP3‐related apoptosis execution. Furthermore, our results implicate YJP in the regulation of the upstream mitochondrial pathway, as kaempferol, quercetin, and wogonin showed relatively favorable predicted binding to the pro‐apoptotic protein BAX (with binding energies of −7.02, −6.40, and −6.37 kcal/mol). This finding is consistent with the network pharmacology prediction of potential modulation of the BCL‐2/BAX regulatory balance and with the established protective role of BCL‐2 [43].

Potential regulation of apoptosis may also involve PI3K‐Akt‐related pro‐survival signaling. The core PPI target AKT1, for which all four components showed predicted binding energies ranging from −6.26 to −6.12 kcal/mol, serves as a key node in the PI3K‐Akt pathway. Since Akt activation promotes cell survival by inhibiting pro‐apoptotic proteins and regulating the BCL‐2 family balance, our data suggest that YJP may employ a dual‐pronged strategy: it may interact with the executioner protein CASP3 while also modulating PI3K‐Akt‐AKT1‐related survival signaling. This coordinated action may influence the BCL‐2/BAX balance and potentially affect the initiation of mitochondrial apoptosis. Collectively, this multi‐tiered model—involving PI3K‐Akt‐related survival signaling, mitochondrial apoptosis regulation, and CASP3‐associated apoptosis execution—provides a testable mechanistic framework for the potential cytoprotective effects of YJP in HT.

### 4.2 Coordination and Integration: From the “Dual‐Axis Core” to Multidimensional Regulation

The “dual‐axis core” model's primary advantage lies in its coordinated targeting of HT's core pathological circuit, potentially offering broader regulatory coverage than single‐target approaches. Complex diseases such as autoimmune disorders often involve network imbalances. While TNF‐α blockers or IKKβ inhibitors may be limited by compensatory pathway activation, and pro‐survival strategies alone may not address inflammatory triggers, YJP's potential parallel modulation of both pathological dimensions provides a bidirectional strategy involving inflammatory regulation and survival/apoptosis‐related processes. This may yield coordinated effects by simultaneously influencing inflammatory signaling and tissue survival‐related pathways.

The “dual‐axis core” model further integrates into a multidimensional network, aligning with TCM's holistic philosophy and systems pharmacology. This coordinated regulation is mechanistically plausible. NF‐κB activation can upregulate pro‐apoptotic factors [44]; therefore, potential modulation of this pathway by YJP may concurrently influence apoptosis initiation. Conversely, CASP3 modulation and potential PI3K‐Akt‐related regulation of thyrocyte survival processes may also influence the local inflammatory microenvironment, for example by reducing cellular damage‐associated signals. These reciprocal interactions may form a mutually reinforcing regulatory framework linking inflammation and apoptosis‐related processes.

Furthermore, KEGG analysis suggests YJP's influence may extend upstream to adaptive immunity, particularly Th17 cell differentiation. Th17/Treg imbalance is central to HT [45, 46], and core targets like STAT3, IKBKB, and RELA are key Th17 regulators. Components such as wogonin have been reported to modulate the Th17/Treg balance [47]. Thus, the “dual‐axis core” model may function as a hub: potentially modulating pathogenic immune cell generation upstream (e.g., Th17/Treg) while potentially influencing downstream tissue‐damage‐related processes through the proposed “inflammation–apoptosis” axis. This multi‐level framework—from immune modulation to cytoprotection—offers a coherent systems biology explanation for YJP's potential in HT.

### 4.3 Study Limitations and Translational Perspectives

This study integrates network pharmacology and molecular docking to construct a “dual‐axis core” model for YJP in treating HT, systematically predicting its polypharmacological mechanism. However, it is important to emphasize that this research is inherently computational and theoretical. Its core findings and mechanistic hypotheses require further experimental validation through systematic in vitro and in vivo studies. Several specific limitations should be noted. First, the TCM component databases used in this study (e.g., TCMSP) are incomplete and may not encompass all active metabolites generated during the in vivo metabolism of the full YJP formula, particularly for Massa Medicata Fermentata (Liushenqu). Consequently, some potential active components and their corresponding targets might have been overlooked. Moreover, as a fermented medicinal material, Liushenqu may exert indirect effects through modulation of the gut microbiota or through fermentation‐ and metabolism‐derived metabolites; future microbiome and metabolomics studies may help characterize these contributions more comprehensively. Second, the relatively favorable docking interactions predicted by molecular docking represent computational potential; the actual binding events within biological systems and their subsequent functional consequences (e.g., agonism or inhibition) must ultimately be verified through experimental assays. Third, molecular dynamics simulations were not performed in the present study; therefore, the temporal stability and conformational dynamics of the predicted ligand–target complexes remain to be further investigated.

Despite these limitations, the principal value of this study lies in providing a molecular roadmap and a set of testable mechanistic hypotheses for advancing YJP from traditional empirical use toward mechanism‐informed therapeutic development. Based on our findings, we propose a stepwise validation and translation strategy from molecular targets to clinical investigation:

#### 4.3.1 Mechanistic Validation at the Cellular Level

In established HT cellular models (e.g., human thyroid follicular cells), the regulatory effects of YJP and its candidate active compounds should be systematically evaluated. This may include assessment of key events in the NF‐κB pathway, such as IKKβ phosphorylation and p65 nuclear translocation, together with the expression of downstream pro‐inflammatory cytokines including TNF‐α and IL‐6. In parallel, the effects of YJP on PI3K‐Akt signaling, the BCL‐2/BAX balance, and CASP3 enzymatic activity should be investigated. Furthermore, gene knockdown or knockout approaches may be used to determine the functional relevance of key targets such as AKT1, RELA, and CASP3 to the observed cellular effects.

#### 4.3.2 Confirmation of Whole‐Formula Effects in Animal Models

In HT animal models (e.g., NOD.H‐2h4 mice), the effects of the whole YJP formula should be evaluated by examining thyroid lymphocytic infiltration, follicular structural damage, and thyroid functional changes. Immunohistochemistry and Western blot analyses may be used to assess relevant molecular markers, such as p‐p65 and cleaved CASP3, and to determine whether their changes are associated with pathological improvement. These experiments would provide in vivo evidence for further evaluation of the proposed “dual‐axis core” model.

#### 4.3.3 Exploration of Clinical Translation and Precision Application

Future clinical studies should preliminarily evaluate the efficacy and safety of YJP as an adjunctive therapy to levothyroxine. Particular attention may be given to changes in thyroid autoantibody levels (TPOAb and TgAb), thyroid function, and patient‐reported quality of life. Potential biomarkers related to the “inflammation–immunity–apoptosis” network identified in this study, such as serum inflammatory cytokine profiles and Th17/Treg cell ratios, may also be incorporated into clinical evaluation. Such studies could help determine the translational relevance of the systems‐level hypotheses proposed here and inform the future clinical evaluation and application of YJP in HT.

## 5 Conclusion

This study applies an integrated computational strategy to investigate the complex polypharmacology of YJP against HT. Through network pharmacology and molecular docking analysis, we predict a ‘multicomponent, multi‐target, multi‐pathway’ mechanism potentially underlying the pharmacological effects of YJP in HT. Our findings provide computational support for a ‘dual‐axis core’ hypothesis in which key YJP constituents—quercetin, luteolin, kaempferol, and wogonin—may coordinately modulate the TNF–NF‐κB inflammatory axis and the PI3K–Akt‐mediated cell survival/apoptosis axis.

Our analysis delineates a coherent dual‐pronged regulatory framework. These components may exert multi‐level modulation of the NF‐κB cascade through key nodes including TNF, IKBKB, RELA, and PTGS2, while also influencing apoptosis‐related signaling through AKT1, the BCL‐2/BAX regulatory balance, and potential interactions with CASP3. Pathway analysis further suggests that this regulatory network may extend to adaptive immune processes, particularly Th17 cell differentiation, linking inflammation, apoptosis, and immune regulation.

Overall, the proposed ‘dual‐axis core’ model provides a testable mechanistic framework for future experimental validation and multi‐target therapeutic research in autoimmune thyroiditis. It also offers a useful systems‐level approach for investigating the pharmacological basis of complex traditional medicine formulations.

## Author Contributions

Conceptualization: **Hao Zhang**, **Kuojun Ren**, and **Shengying Wang**. Writing – original draft: **Hao Zhang**. Writing – review and editing: **Kuojun Ren** and **Shengying Wang**.

## Funding

This research was supported by the Anhui Provincial Clinical Medicine Research Transformation Project of 2025 (202527c10020112) and the Anhui Provincial Health and Health Research Project (AHWJ2023BAa20166).

## Ethics Statement

The authors have nothing to report.

## Consent

Consent for publication is not applicable to this study, as no individual patient data, personal information, or identifiable information were included.

## Conflicts of Interest

The authors declare no conflicts of interest.

## Use of Generative AI and AI‐Assisted Technologies in the Writing Process

ChatGPT (OpenAI) was used solely for English language editing, including grammar, tense, and sentence clarity.

## Trial Registration

Trial registration is not applicable because this study did not involve human participants or a clinical trial.

## Supporting information




**Supporting file 1**: cbdv71790‐sup‐0001‐TableS1.docx

**Supporting file 2**: cbdv71790‐sup‐0002‐TableS2.docx

**Supporting file 3**: cbdv71790‐sup‐0003‐TableS3.docx

**Supporting file 4**: cbdv71790‐sup‐0004‐TableS4.docx

**Supporting file 5**: cbdv71790‐sup‐0005‐TableS5.docx

## Data Availability

The data that support the findings of this study are available from the corresponding author upon reasonable request.
