# Supplementary material for: Coordinated TNF–NF‐κB and PI3K–Akt Modulation by Yueju Pill in Hashimoto's Thyroiditis: A Network Pharmacology and Molecular Docking Study
Source: Chem Biodivers. 2026 Sep 30;23(10):e71790. doi: 10.1002/cbdv.71790 (PMC13628139; doi:10.1002/cbdv.71790)
Supplement: Supplementary file 1 [file CBDV-23-e71790-s001.docx]

**Supplementary Table S4. Docking scores of reference / positive-control ligands alongside the four YJP active components**

| Target (PDB ID) | Reference ligand (type) | Reference docking score (kcal/mol) | quercetin | luteolin | kaempferol | wogonin |
| --- | --- | --- | --- | --- | --- | --- |
| CASP3 (1GFW) | Isatin sulfonamide (MSI) — co-crystal ligand | -8.77 | -6.91 | -7.81 | -7.26 | -7.61 |
| IKBKB (4KIK) | SD4 inhibitor (KSA) — co-crystal ligand | -13.91 | n.d. | n.d. | -8.20 | n.d. |
| MAPK1 (4QTA) | SCH772984 (38Z) — co-crystal ligand | -12.46 | -9.47 | -9.68 | n.d. | n.d. |
| AKT1 (1H10) | Ins(1,3,4,5)P4 (4IP) — co-crystal ligand | -6.55 | -6.17 | -6.26 | -6.25 | -6.12 |
| PTGS2 (5F19) | Celecoxib — known COX-2 inhibitor | -9.18 | -8.33 | -9.02 | -8.87 | -8.88 |
| TNF (1TNF) | SPD304 — known TNF-α inhibitor | -9.88 | -9.18 | -9.23 | -9.20 | -8.65 |
| BAX (4BD6) | BAM7 — known BAX-binding ligand | -5.87 | -6.40 | n.d. | -7.02 | -6.37 |
| IL6 (1ALU) | Madindoline A — known IL-6 inhibitor | -6.20 | -6.37 | -7.12 | n.d. | -6.39 |
| RELA (3QXY) | S-adenosylmethionine (SAM) — co-crystallized cofactor | -7.30 | -9.17 | -9.01 | -9.20 | -9.01 |

Note: Reference ligands were docked under the same AutoDock Vina v1.2.7 protocol as the YJP compounds (grid spacing 0.375 Å, exhaustiveness = 8, box size 30 × 30 × 30 Å; grid box parameters from Supplementary Table S2). For targets with a co-crystallized small-molecule ligand (CASP3, IKBKB, MAPK1, AKT1, RELA), the co-crystal ligand itself served as the reference (redocking RMSD values are given in Supplementary Table S3). For targets lacking a redockable small-molecule co-crystal ligand (TNF, PTGS2, BAX, IL6), a known small-molecule inhibitor/antagonist was used as a positive control: SPD304 (TNF-α inhibitor), celecoxib (COX-2 inhibitor), BAM7 (a BAX-binding ligand; BAX lacks a canonical small-molecule inhibitor), and madindoline A (IL-6 inhibitor). For RELA, the co-crystallized cofactor SAM was redocked at its cofactor-binding site, which is distinct from the YJP-docked cavity; the comparison is therefore site-specific. Docking scores are comparative computational indicators (the absolute accuracy of AutoDock Vina scores is approximately 2 kcal/mol) rather than direct measurements of binding affinity; the four YJP compounds showed predicted binding energies comparable to—and for several targets more favorable than—the reference / known-inhibitor ligands, supporting a comparative rather than absolute-threshold interpretation of the docking results (see response to Comment 15). n.d. = not docked in the original study.
