# Supplementary material for: Coordinated TNF–NF‐κB and PI3K–Akt Modulation by Yueju Pill in Hashimoto's Thyroiditis: A Network Pharmacology and Molecular Docking Study
Source: Chem Biodivers. 2026 Sep 30;23(10):e71790. doi: 10.1002/cbdv.71790 (PMC13628139; doi:10.1002/cbdv.71790)
Supplement: Supplementary file 2 [file CBDV-23-e71790-s002.docx]

**Supplementary Table S2. Grid box parameters used for molecular docking of active compounds with target proteins**

| **Target** | **Compound** | **PDB ID** | **Center (x, y, z)** | **Grid size (x, y, z)** |
| --- | --- | --- | --- | --- |
| BAX | Kaempferol | 4BD6 | -34.332, 9.099, 0.923 | 30 × 30 × 30 Å |
| BAX | Quercetin | 4BD6 | -14.854, -28.084, -16.257 | 30 × 30 × 30 Å |
| BAX | Wogonin | 4BD6 | -14.854, -28.084, -16.257 | 30 × 30 × 30 Å |
| AKT1 | Kaempferol | 1H10 | 16.782, 5.399, 13.553 | 30 × 30 × 30 Å |
| AKT1 | Luteolin | 1H10 | 16.782, 5.399, 13.553 | 30 × 30 × 30 Å |
| AKT1 | Quercetin | 1H10 | 16.782, 5.399, 13.553 | 30 × 30 × 30 Å |
| AKT1 | Wogonin | 1H10 | 15.294, 20.969, 15.904 | 30 × 30 × 30 Å |
| CASP3 | Kaempferol | 1GFW | 37.226, 34.672, 31.716 | 30 × 30 × 30 Å |
| CASP3 | Luteolin | 1GFW | 42.069, 24.224, 25.582 | 30 × 30 × 30 Å |
| CASP3 | Quercetin | 1GFW | 21.082, 6.992, 34.997 | 30 × 30 × 30 Å |
| CASP3 | Wogonin | 1GFW | 37.226, 34.672, 31.716 | 30 × 30 × 30 Å |
| IKBKB | Kaempferol | 4KIK | 53.661, 2.988, -51.395 | 30 × 30 × 30 Å |
| IL6 | Luteolin | 1ALU | 0.128, -23.190, -2.198 | 30 × 30 × 30 Å |
| IL6 | Quercetin | 1ALU | 5.783, -24.221, 17.667 | 30 × 30 × 30 Å |
| IL6 | Wogonin | 1ALU | 5.783, -24.221, 17.667 | 30 × 30 × 30 Å |
| MAPK1 | Luteolin | 4QTA | 28.470, 55.504, 35.798 | 30 × 30 × 30 Å |
| MAPK1 | Quercetin | 4QTA | 28.470, 55.504, 35.798 | 30 × 30 × 30 Å |
| PTGS2 | Kaempferol | 5F19 | 21.578, 39.099, 35.962 | 30 × 30 × 30 Å |
| PTGS2 | Luteolin | 5F19 | 21.578, 39.099, 35.962 | 30 × 30 × 30 Å |
| PTGS2 | Quercetin | 5F19 | 13.702, 48.654, 64.724 | 30 × 30 × 30 Å |
| PTGS2 | Wogonin | 5F19 | 31.625, 37.475, 17.620 | 30 × 30 × 30 Å |
| RELA | Kaempferol | 3QXY | 61.418, 19.400, 8.547 | 30 × 30 × 30 Å |
| RELA | Luteolin | 3QXY | 61.418, 19.400, 8.547 | 30 × 30 × 30 Å |
| RELA | Quercetin | 3QXY | 61.418, 19.400, 8.547 | 30 × 30 × 30 Å |
| RELA | Wogonin | 3QXY | 61.418, 19.400, 8.547 | 30 × 30 × 30 Å |
| TNF | Kaempferol | 1TNF | 8.255, 62.605, 31.084 | 30 × 30 × 30 Å |
| TNF | Luteolin | 1TNF | 8.255, 62.605, 31.084 | 30 × 30 × 30 Å |
| TNF | Quercetin | 1TNF | 8.255, 62.605, 31.084 | 30 × 30 × 30 Å |
| TNF | Wogonin | 1TNF | 8.255, 62.605, 31.084 | 30 × 30 × 30 Å |
