# Supplementary material for: Coordinated TNF–NF‐κB and PI3K–Akt Modulation by Yueju Pill in Hashimoto's Thyroiditis: A Network Pharmacology and Molecular Docking Study
Source: Chem Biodivers. 2026 Sep 30;23(10):e71790. doi: 10.1002/cbdv.71790 (PMC13628139; doi:10.1002/cbdv.71790)
Supplement: Supplementary file 3 [file CBDV-23-e71790-s003.docx]

**Supplementary Table S1. Information of protein structures used for molecular docking**

| **Target** | **Protein name** | **PDB ID** | **Chain** | **Resolution** | **Organism** | **Native ligand** |
| --- | --- | --- | --- | --- | --- | --- |
| BAX | Apoptosis regulator BAX | 4BD6 | A | 2.49 | Homo sapiens | BAX BH3 peptide |
| CASP3 | Caspase-3 (apopain) | 1GFW | A/B | 2.8 | Homo sapiens | Isatin sulfonamide inhibitor |
| IKBKB | Inhibitor of nuclear factor kappa-B kinase subunit beta (IKKβ) | 4KIK | A | 2.83 | Homo sapiens | SD4 inhibitor |
| IL6 | Interleukin-6 | 1ALU | A | 1.9 | Homo sapiens | None |
| MAPK1 | Mitogen-activated protein kinase 1 (ERK2) | 4QTA | A | 1.45 | Homo sapiens | SCH772984 |
| PTGS2 | Prostaglandin G/H synthase 2 (COX-2) | 5F19 | A/B | 2.04 | Homo sapiens | Acetylated Ser530 (aspirin-modified COX-2) |
| TNF | Tumor necrosis factor-alpha | 1TNF | A/B/C | 2.6 | Homo sapiens | None |
| AKT1 | RAC-alpha serine/threonine kinase (AKT1) PH domain | 1H10 | A | 1.4 | Homo sapiens | Ins(1,3,4,5)-tetrakisphosphate |
| RELA | NF-κB p65 (RELA) peptide fragment in complex with SETD6 | 3QXY | P/Q | 2.09 | Homo sapiens | NF-κB p65 (RELA) peptide fragment |
