# Supplementary material for: Coordinated TNF–NF‐κB and PI3K–Akt Modulation by Yueju Pill in Hashimoto's Thyroiditis: A Network Pharmacology and Molecular Docking Study
Source: Chem Biodivers. 2026 Sep 30;23(10):e71790. doi: 10.1002/cbdv.71790 (PMC13628139; doi:10.1002/cbdv.71790)
Supplement: Supplementary file 4 [file CBDV-23-e71790-s004.docx]

**Supplementary Table S5A. Key molecular interactions between YJP-derived active compounds and core target proteins identified by molecular docking**

| Target | Compound | Hydrogen bonds | Hydrophobic contacts | π–π stacking | Salt bridges |
| --- | --- | --- | --- | --- | --- |
| AKT1 | Kaempferol | HIS13A, GLU95A | TRP11A, PRO24A | TRP11A, HIS13A | — |
| AKT1 | Luteolin | LYS20A, THR65A, ARG67A, GLU17A, ARG15A, GLY16A | ILE74A | — | — |
| AKT1 | Quercetin | TRP11A, GLU95A, LYS8A, GLU9A, TRP99A, HIS89A, GLU91A | TRP11A, VAL90A | — | — |
| AKT1 | Wogonin | GLN47A, ALA50A, LEU52A | TYR38A, LEU52A | — | — |
| BAX | Kaempferol | LEU161A, GLN155A, GLY160A | TRP151A, LEU161A, TYR164A, TRP158A | TRP151A, TYR164A | — |
| BAX | Quercetin | ARG94A, THR56C, ALA54C, SER55C, SER87A | VAL91A, VAL83A, ALA54C | — | — |
| BAX | Wogonin | ALA117A, LEU120A, ARG89A | LEU120A, PHE92A, PRO88A, ALA117A, LEU27A | PHE92A | — |
| CASP3 | Kaempferol | ASP253B, ARG207B, SER205B, TRP206B | TRP206B, TYR204B, PHE256B | TRP206B, PHE256B | — |
| CASP3 | Luteolin | GLU248B, PHE250B, GLU246B, ASP211B, GLN217B | PHE247B, TRP214B, PHE250B, TRP206B | TRP214B, PHE247B | — |
| CASP3 | Quercetin | HIS277B, PHE275B, LEU223B, LYS224B, ALA227B, ASP228B | LEU81A, ALA227B, TYR276B, MET44A | — | — |
| CASP3 | Wogonin | SER205B, ARG207B | TYR204B, PHE256B, TRP206B | TYR204B, TRP206B, PHE256B | — |
| IKBKB | Kaempferol | GLY218A, ARG427A, TYR571A, ARG220A, ASN225A, LYS428A, GLN432A | PHE219A, PRO224A | — | — |
| IL-6 | Luteolin | ARG30A, ASP34A, LEU33A, GLN175A, ARG179A | LEU178A, LEU33A | — | — |
| IL-6 | Quercetin | ASN61A, ASN63A, LYS150A, GLU93A, ASN144A | LEU147A, VAL96A, TYR97A, PRO139A | — | — |
| IL-6 | Wogonin | THR137A, THR138A | PRO139A, VAL96A, TYR97A | — | — |
| MAPK1 | Luteolin | LYS54A, ASP167A | TYR36A, ILE56A, ILE103A, VAL39A | TYR36A | — |
| MAPK1 | Quercetin | LYS54A, ASP167A | TYR36A, ILE56A, ILE103A, VAL39A | TYR36A | — |
| PTGS2 | Kaempferol | ASN375A, TYR373A, GLN374B, VAL228A, GLY533A, ASN537A, ARG376A | PHE142B, TRP139B | — | — |
| PTGS2 | Luteolin | GLY225B, VAL228B, ASN537B, GLY533B, SER143A | PHE142A, LEU145A, TRP139A | PHE142A | — |
| PTGS2 | Quercetin | SER126A, ARG44A, HIS122A, ILE124A, GLN372A, LYS532A, THR118A, GLN370A | PHE371A, PRO542B | HIS122A | — |
| PTGS2 | Wogonin | THR206B, TYR385B, ALA202B, ALA199B | ALA202B, TYR385B, VAL447B, LEU391B | HIS207B, HIS386B | — |
| RELA | Kaempferol | ASN251A, HIS252A, TYR285A, TYR223A | VAL72A, ALA73A, PHE299A, TYR285A, TYR223A, ALA222A | PHE299A | — |
| RELA | Luteolin | ASN251A, HIS252A, TYR285A | ALA73A, PHE299A, VAL72A, TYR285A, LEU253A | PHE299A | — |
| RELA | Quercetin | ASN251A, HIS252A, TYR285A, TYR223A | VAL72A, ALA73A, PHE299A, TYR285A, TYR223A, ALA222A | PHE299A | — |
| RELA | Wogonin | ASN251A, HIS252A, TYR285A | ALA73A, PHE299A, VAL72A, LEU253A | PHE299A | — |
| TNF | Kaempferol | GLN102B, PRO100C, GLU116C, SER99A, GLU116B | — | — | — |
| TNF | Luteolin | GLN102B, TYR115A, GLU116A, SER99C, PRO100B, GLN102A | TRP114A | — | — |
| TNF | Quercetin | GLN102B, PRO100C, ARG103B, GLU116C, SER99A, GLU116B | — | — | — |
| TNF | Wogonin | PRO100B, GLN102B, SER99B, SER99C | — | — | — |

Note: Interactions were computed from the lowest-energy docking pose (result.pdb) of each compound–target pair. Hydrogen bonds: ligand N/O/S atoms within 3.5 Å of receptor N/O/S atoms (heavy-atom cutoff). Hydrophobic contacts: ligand carbon within 4.5 Å of side-chain carbons of hydrophobic residues. π–π stacking: ligand aromatic ring centroid within 5.5 Å of an aromatic (Phe/Tyr/Trp/His) ring centroid; P = near-parallel, T = T-shaped, I = intermediate. Salt bridges: oppositely charged ligand and receptor groups within 4.5 Å. Residues are labelled as residue-type + residue-number + chain. AutoDock Vina v1.2.7 / PyMOL-derived docking poses; interaction geometry computed with RDKit.

**Supplementary Table S5B. Comparison of binding-site residues contacted by YJP-derived active compounds with those contacted by the native / co-crystallized ligand**

| Target | Native ligand | Native-ligand contacting residues | YJP-compound contacting residues (union) | Shared with native |
| --- | --- | --- | --- | --- |
| CASP3 | MSI (isatin sulfonamide) | ARG207B, CYS163A, GLY122A, HIS121A, MET61A, PHE256B, SER205B, TRP206B, TYR204B | ALA227B, ARG207B, ASP211B, ASP228B, ASP253B, GLN217B, GLU246B, GLU248B, HIS277B, LEU223B, LEU81A, LYS224B, MET44A, PHE247B, PHE250B, PHE256B, PHE275B, SER205B, TRP206B, TRP214B, TYR204B, TYR276B | ARG207B, PHE256B, SER205B, TRP206B, TYR204B |
| IKBKB | KSA (SD4 inhibitor) | ALA42A, CYS99A, GLU149A, GLU97A, ILE165A, LEU21A, MET96A, TYR98A, VAL152A, VAL29A, VAL74A | ARG220A, ARG427A, ASN225A, GLN432A, GLY218A, LYS428A, PHE219A, PRO224A, TYR571A | — |
| MAPK1 | SCH772984 (38Z) | ALA35A, ALA52A, ASP106A, GLN105A, ILE31A, ILE56A, ILE84A, LEU107A, LEU156A, LYS114A, LYS54A, MET108A, PRO58A, TYR36A, TYR64A, VAL39A | ASP167A, ILE103A, ILE56A, LYS54A, TYR36A, VAL39A | ILE56A, LYS54A, TYR36A, VAL39A |
| AKT1 | Ins(1,3,4,5)P4 (4IP) | ARG23A, ARG25A, ARG86A, ASN53A, GLU17A, ILE19A, LYS14A, TYR18A | ALA50A, ARG15A, ARG67A, GLN47A, GLU17A, GLU91A, GLU95A, GLU9A, GLY16A, HIS13A, HIS89A, ILE74A, LEU52A, LYS20A, LYS8A, PRO24A, THR65A, TRP11A, TRP99A, TYR38A, VAL90A | GLU17A |
| RELA | SAM (cofactor) | ALA73A, ASN251A, EDO480A, HIS252A, PHE299A, TYR223A, TYR285A, TYR297A, TYR75A, VAL72A | ALA222A, ALA73A, ASN251A, HIS252A, LEU253A, PHE299A, TYR223A, TYR285A, VAL72A | ALA73A, ASN251A, HIS252A, PHE299A, TYR223A, TYR285A, VAL72A |

Note: For the five targets with a redockable co-crystallized small-molecule ligand, the residues contacted by the native ligand (computed from the crystal structure) were compared with the union of residues contacted by the four YJP compounds. Shared residues indicate that the YJP compounds engage the same binding-site residues as the experimentally validated ligand, supporting the biological plausibility of the predicted binding modes.
