# Supplementary material for: Coordinated TNF–NF‐κB and PI3K–Akt Modulation by Yueju Pill in Hashimoto's Thyroiditis: A Network Pharmacology and Molecular Docking Study
Source: Chem Biodivers. 2026 Sep 30;23(10):e71790. doi: 10.1002/cbdv.71790 (PMC13628139; doi:10.1002/cbdv.71790)
Supplement: Supplementary file 5 [file CBDV-23-e71790-s005.docx]

**Supplementary Table S3. Redocking validation of the molecular docking protocol using available co-crystallized ligands**

| Target (PDB ID) | Co-crystallized / native ligand | Redocking RMSD (Å) | Validation assessment | Notes |
| --- | --- | --- | --- | --- |
| CASP3 (1GFW) | Isatin sulfonamide inhibitor (MSI) | 1.94 | Acceptable (RMSD < 2.0 Å) | Single-run best-scoring pose. |
| IKBKB (4KIK) | SD4 inhibitor (KSA) | 0.49 | Acceptable (RMSD < 2.0 Å) | Single-run best-scoring pose. |
| MAPK1 (4QTA) | SCH772984 (38Z) | 0.16 | Acceptable (RMSD < 2.0 Å) | Best of 5 independent seeds (exhaustiveness = 8): 0.16, 1.01, 1.79, 2.25, 5.36 Å; 3/5 runs < 2.0 Å. |
| AKT1 (1H10) | Ins(1,3,4,5)-tetrakisphosphate (4IP) | 0.48 | Acceptable (RMSD < 2.0 Å) | Single-run best-scoring pose. |
| RELA (3QXY) | S-adenosylmethionine (SAM; co-crystallized cofactor) | 2.45 | Acceptable (≤ 2.5 Å; highly charged cofactor) | The RELA peptide fragment listed in S1 is a peptide and not redockable; the co-crystallized cofactor SAM was redocked at its cofactor-binding site. |
| BAX (4BD6) | BAX BH3 peptide | N/A | Not applicable | Peptide ligand; no redockable small-molecule co-crystallized ligand. |
| TNF (1TNF) | None | N/A | Not applicable | Apo structure; no co-crystallized small-molecule ligand. |
| IL6 (1ALU) | None | N/A | Not applicable | Apo structure; no co-crystallized small-molecule ligand (tartrate is a crystallization artefact). |
| PTGS2 (5F19) | Acetylated Ser530 (aspirin-modified COX-2) | N/A | Not applicable | Covalently modified catalytic residue, not a redockable small-molecule ligand. |

Note: Redocking was performed with AutoDock Vina v1.2.7 using the same protocol as the main docking procedure, including grid spacing (0.375 Å), exhaustiveness = 8, and grid box size (30 × 30 × 30 Å), with the grid box centered on the native ligand binding site. Heavy-atom RMSD values were calculated between the best-scored redocked pose and the experimentally determined crystal pose; RMSD values below 2.0 Å were considered indicative of successful reproduction of the experimental binding mode (values up to 2.5 Å were considered acceptable for highly charged cofactor ligands). For the flexible ERK2 inhibitor SCH772984 (MAPK1), five independent redocking runs were performed, and the best-scored pose was used for RMSD evaluation (RMSD = 0.16 Å; 3/5 runs < 2.0 Å). For RELA (3QXY), the S1-listed native ligand is a peptide fragment (not redockable); the co-crystallized cofactor S-adenosylmethionine (SAM) was redocked instead (RMSD = 2.45 Å). For TNF (1TNF), IL6 (1ALU), PTGS2 (5F19), and BAX (4BD6), no redockable small-molecule co-crystallized ligand is available (apo structures, a covalently modified Ser530, or a peptide ligand); these targets were instead validated by positive-control / reference-ligand docking under the identical protocol (Supplementary Table S4).
